# Open-source discovery of chemical leads for next-generation chemoprotective antimalarials

**DOI:** 10.1126/science.aat9446

**Published:** 2018-12-07

**Authors:** Yevgeniya Antonova-Koch, Stephan Meister, Matthew Abraham, Madeline R. Luth, Sabine Ottilie, Amanda K. Lukens, Tomoyo Sakata-Kato, Manu Vanaerschot, Edward Owen, Juan Carlos Jado, Steven P. Maher, Jaeson Calla, David Plouffe, Yang Zhong, Kaisheng Chen, Victor Chaumeau, Amy J. Conway, Case W. McNamara, Maureen Ibanez, Kerstin Gagaring, Fernando Neria Serrano, Korina Eribez, Cullin McLean Taggard, Andrea L. Cheung, Christie Lincoln, Biniam Ambachew, Melanie Rouillier, Dionicio Siegel, François Nosten, Dennis E. Kyle, Francisco-Javier Gamo, Yingyao Zhou, Manuel Llinás, David A. Fidock, Dyann F. Wirth, Jeremy Burrows, Brice Campo, Elizabeth A. Winzeler

**Affiliations:** 1 School of Medicine, University of California, San Diego, 9500 Gilman Drive 0760, La Jolla, CA 92093, USA; 2 Harvard T. H. Chan School of Public Health, 665 Huntington Avenue, Boston, MA 02115, USA; 3 The Broad Institute, 415 Main Street, Cambridge, MA 02142, USA; 4 Division of Infectious Diseases, Department of Microbiology and Immunology, Columbia University Medical Center, New York, NY 10032, USA; 5 Department of Biochemistry and Molecular Biology and Center for Malaria Research, Pennsylvania State University, University Park, PA 16802, USA; 6 Center for Tropical and Emerging Global Diseases, University of Georgia, 500 D. W. Brooks Drive, Athens, GA 30602, USA; 7 Department of Global Health, University of South Florida, 3720 Spectrum Boulevard, Tampa, FL 33612, USA; 8 The Genomics Institute of the Novartis Research Foundation, 10675 John J Hopkins Drive, San Diego, CA 92121, USA; 9 Shoklo Malaria Research Unit, Mahidol Oxford Research Unit, Faculty of Tropical Medicine, Mahidol University, Mae Sot, Thailand; 10 Centre for Tropical Medicine and Global Health, Nuffield Department of Medicine, University of Oxford, Oxford, UK; 11 Tres Cantos Medicines Development Campus, Malaria DPU, GlaxoSmithKline, Severo Ochoa 2, Tres Cantos 28760, Madrid, Spain; 12 Medicines for Malaria Venture, Post Office Box 1826, 20 Route de Pre-Bois, 1215 Geneva 15, Switzerland; 13 Skaggs School of Pharmacy and Pharmaceutical Sciences, University of California, San Diego, 9500 Gilman Drive 0741, La Jolla, CA 92093, USA; 14 Department of Chemistry and Center for Infectious Diseases Dynamics, Pennsylvania State University, University Park, PA 16802, USA

## Abstract

To discover leads for next-generation chemoprotective antimalarial drugs,we tested more than 500,000 compounds for their ability to inhibit liver-stage development of luciferase-expressing *Plasmodium* spp. parasites (681 compounds showed a half-maximal inhibitory concentration of less than 1micromolar).Cluster analysis identified potent and previously unreported scaffold families as well as other series previously associated with chemoprophylaxis. Further testing through multiple phenotypic assays that predict stage-specific and multispecies antimalarial activity distinguished compound classes that are likely to provide symptomatic relief by reducing asexual blood-stage parasitemia from those which are likely to only prevent malaria. Target identification by using functional assays, in vitro evolution, or metabolic profiling revealed 58 mitochondrial inhibitors but also many chemotypes possibly with previously unidentified mechanisms of action.

**INTRODUCTION:**

Malaria remains a devastating disease, affecting 216 million people annually, with 445,000 deaths occurring primarily in children under 5 years old. Malaria treatment relies primarily on drugs that target the diseasecausing asexual blood stages (ABS) of *Plasmodium* parasites, the organisms responsible for human malaria. Whereas travelers may rely on shortterm daily chemoprotective drugs, those living in endemic regions require long-termmalaria protection such as insecticide-treated nets (ITNs) and vector control. However, ITNs do not fully shield individuals from malaria, may lose potency with time, and can be bulky and difficult to use. Another concern is that mosquitosmay become resistant to the active insecticides that are used in ITNs and vector control.

**RATIONALE:**

As the possibility of malaria elimination becomesmore tangible, the ideal antimalarial medicine profile should include chemoprotection. Chemoprotectivemedicines typically work against the exoerythrocytic parasite forms that invade and develop in the liver and are responsible for the earliest asymptomatic stage of the infection. Such medicines could be formulated to provide long-acting prophylaxis, safeguarding individuals that are living near or traveling to areas that have been cleared of parasites. Long-acting chemoprotection in endemic regions could also greatly reduce circulating parasite numbersandpotentially replace a vaccine in an elimination campaign. Although millions of compounds have been screened for activity against parasiteABS, and some have been subsequently tested for potential prophylactic activity, large-scale searches that beginwith prophylactic activity have not been performed because of the complexity of the assay: This assay requires the production of infected laboratory-rearedmosquitoes and hand-dissection of the sporozoiteinfected salivary glands frommosquito thoraxes.

**RESULTS:**

To discover leads for next-generation chemoprotective antimalarial drugs, we used luciferase-expressing *Plasmodium* spp. parasites, dissected from more than a million mosquitoes over a 2-year period, to test more than 500,000 compounds for their ability to inhibit liver-stage development of malaria (681 compounds showed a half-maximal inhibitory concentration of<1 µM). Cluster analysis identified potent and previously unreported scaffold families, as well as other series previously associatedwith chemoprophylaxis. These leads were further tested through multiple phenotypic assays that predict stagespecific and multispecies antimalarial activity. This work revealed compound classes that are likely to provide symptomatic relief from bloodstage parasitemia in addition to providing protection. Target identification by use of functional assays, in vitro evolution, or metabolic profiling revealed 58 mitochondrial inhibitors but also many chemotypes possibly with previously unknownmechanisms of action, somewhichmay disrupt the host pathogen signaling.

**CONCLUSION:**

Our data substantially expands the set of compounds with demonstrated activity against two known targets of chemoprotective drugs, cytochrome bc1 and dihydroorotate dehydrogenase. These present a rich collection of chemical diversity that may be exploited by members of the community seeking to accelerate malaria elimination with chemoprotection and chemoprophylaxis through open-source drug discovery.

**Figure:**
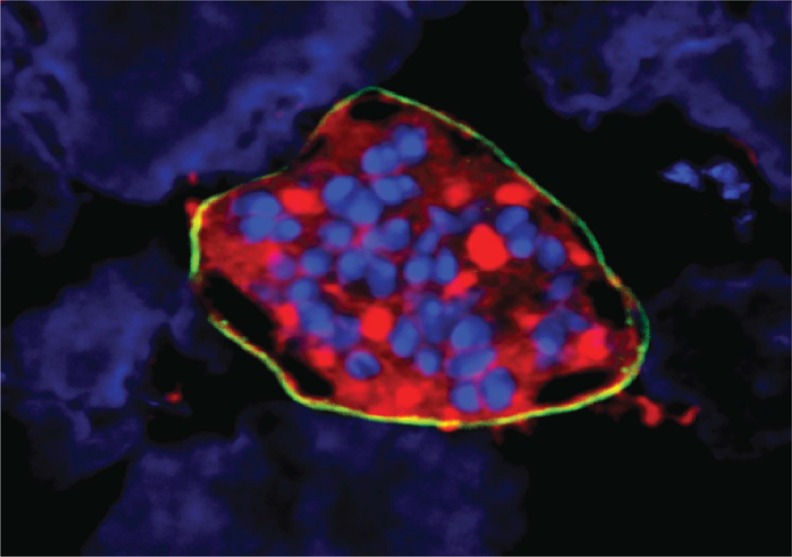
**A *Plasmodium vivax* liver-stage schizont on a lawn of hepatocytes.** The parasite schizont has been stained with antibodies to parasite HSP70 (red) and UIS4 (yellow). Cell (parasite and hepatoma) nuclei are shown in blue. This study identifies compounds that can prevent the development of these liver-stage parasites and may function as chemoprotective drugs for malaria.

Malaria remains a devastating disease, with 216 million annual cases and 445,000 deaths, primarily in children under 5 years old. Malaria treatment relies primarily on drugs that target the disease-causing asexual blood stages (ABS) of the parasite *Plasmodium falciparum.* These drugs include the 4-aminoquinolines piperaquine and amodiaquine, the antifolates pymimethamine and sulfadoxine, and the endoperoxides artemisinin and its derivatives artesunate, artemether, and dihydroartemisinin (*1*). Artemisinin-based combination therapies (ACTs, such as artemetherlumefantrine) are being used worldwide as first-line treatments (*1*).

Although estimated malaria mortality rates have decreased by 47% worldwide since 2000 (*1*), in Southeast Asia resistance has emerged to the artemisinins, and ACT treatment failures are rising (*2*). In anticipation of eventual widespread ACT failure, there has been a focused and coordinated effort to place new antimalarial candidates into the drug development pipeline (www.mmv.org/research-development/rd-portfolio) (*3*). However, in vitro resistance can be generated for most of these new classes in ABS parasites (*4*), suggesting that the antimalarial pipeline will require continuous replenishment.

An alternative approach is to create drugs that prevent malaria by inhibiting parasites during their initial stage of development in the liver before their initiation of symptomatic blood-stage infection. Using chemotherapy for prophylaxis is a well-established tool to prevent malaria caused by different Plasmodiumspp. To preventmalaria, travelersmay take oral atovaquone plus proguanil, doxycycline, or mefloquine. In endemic regions of seasonal malaria in West Africa, children are given SPAQ [sulphadoxine-pyrimethamine (SP) plus amodiaquine] as seasonal malaria chemoprevention, and pregnant women, who are the most vulnerable group, may also take SP for intermittent preventative therapy (*5*).

Drugs that selectively affect the developing liver stages of P. falciparum and the relapsing species, *Plasmodium* vivax, have the potential to engage new protein targets not present nor required in parasite blood stages. Such drugs could overcome both the problem of resistance and compliance. The number of parasites in the early liver stage are low (hundreds, versus billions in the blood stage), reducing the probability that drug resistance–conferring mutations might emerge. This feature could make these liver-active compounds suitable for chemoprotection and, with sufficient demonstrated safety, for mass drug-administration or malaria-elimination campaigns.

To identify chemoprotective candidates, we applied a liver-stage phenotypic screen to a library of >500,000 small molecules. Our data identify new scaffold families that exclusively target liver stages that may provide prophylactic protection, as well as new scaffolds that act against known targets such as dihydroorotate dehydrogenase (DHODH). These data comprise new leads for antimalarial open-source drug discovery.

## Primary screening results

Previous high-throughput screens for antimalarial compounds have generally focused on the ABS, which can be readily cultured en masse (*6–8*). To discover hits with possible protective activity, blood stage–active compounds have been further retested against malaria hepatic stages, often using sporozoites from the rodent malaria species *Plasmodium yoelii* or *Plasmodium berghei* (*9**,* *10*). Because there are no suitable methods for axenically culturing sporozoites (which are responsible for liver-stage infection), this retesting has required the production of infected laboratoryreared mosquitoes and hand dissection of the sporozoite-infected salivary glands from mosquito thoraxes. Despite this complex challenge, thousands of compounds have been examined by using this general workflow progression, leading to previously unidentified chemoprotective candidates, including KAF156 (*11*), KDU691 (*12*), DDD107498 (*13*), and BRD3444 (*14*). However, this approach would not reveal compounds that might protect from infection without affecting blood stages nor identify compounds that modulate human hepatocyte targets and prevent parasite liver-stage development.

Recently, a screening method was developed that was suitable for testing many compounds against liver-stage parasites (*10*). This assay (*Pbluc*) relies on an antifolate-resistant *P. berghei* rodent parasite that constitutively expresses luciferase during the sporozoite stage (*15*). Sporozoites are dissected frommosquitoes, washed, and layered on confluent HepG2 cells that have been preincubated with compounds. If the parasites are able to establish a successful exoerythrocytic stage infection, even if only 1 to 2% of the hepatocytes are infected, the cultures will emit enough light in the presence of luciferin to be detected with sensitive instruments. Light intensity is proportional to parasite infection rate, and this simple assay can be run by using 1536-well plates. The *P. berghei* system has a distinct advantage over the human pathogen, *P. falciparum*, in that this parasite can infect a variety of hepatoma cell lines, which results in ease of use and reproducibility. Furthermore, the development period is only 48 hours, which means that hepatocyte cultures do not overgrow, and special coculture systems are not needed. Sporozoite yields are generally higher, and there are fewer biosafety precautions. In addition, because cellular detoxification systems have been generally lost from hepatoma cell lines, compounds remain potent, easing their discovery. The assay has shown to be very predictive of causal prophylactic activity. We thus sought to apply this screening method to a large library, with the goal of finding a larger selection of starting points for chemoprophylactic drugs.

Screening was conducted by using a library of 538,273 compounds from Charles River consisting of small moleculeswith an averageweight of 369 Da (60.06 to 1298 Da). The library was mostly drug-like (versus probe-like), with an average logP of 3, an average number of hydrogen bond donors of 1.1, and 5.9 hydrogen bond acceptors. On average, each compound had 6.2 rotatable bonds; 5270 compounds were represented in duplicate or triplicate (table S1). Substantial structural redundancy was included in the library to establish structure-activity relationships in scaffold families. In contrast to many previously published studies (*6–8*), all the compounds in the library, including hits and negatives, have structures and are commercially available, facilitating use by the community.

**Fig. 1:**
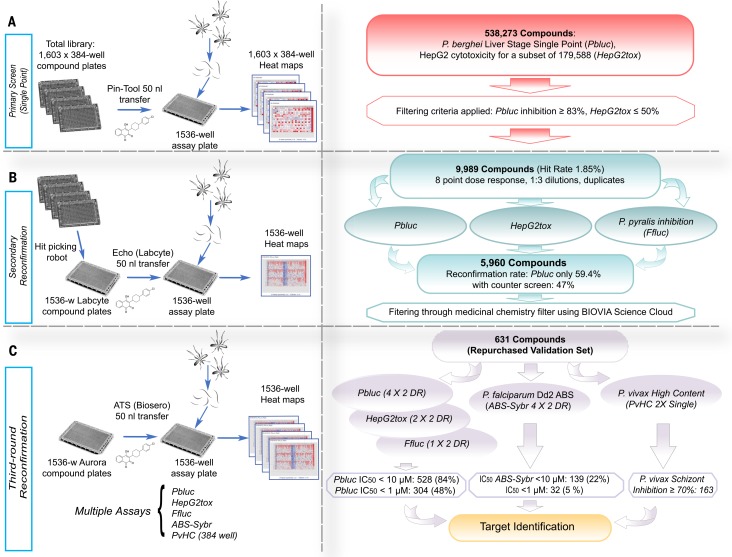
**Screen workflow.** (**A**) The Charles River library (538,273 compounds) was plated into 1603 384-well plates. For the primary *Pbluc* single-point screen, compounds from four of the 384-well plates were transferred with a pin-tool instrument (50 nl per well) into 1536-well assay plates containing seeded HepG2 cells (3 × 103 cells per well).The next day, *P. berghei* luciferase–expressing sporozoites were freshly prepared from infected Anopheles stephensi mosquitoes, and ~1000 sporozoites in a 5 ml volume were added to each well. After 48 hours, *P. berghei*–Luc growth within hepatocytes was measured with bioluminescence. (**B**) To prepare source plates for the first round of reconfirmation (second round of screening), the 9989 hit compounds were transferred from the original 384-well library with an automated hit-picking system and serially diluted into eight points (1:3 dilutions) for dose-response screening in duplicate. The hit compounds (50 nl per well) in serial dilutions were acoustically transferred into assay wells containing HepG2 cells for *Pbluc* and *HepG2tox* assays. In addition, biochemical recombinant luciferase inhibition assay (Ffluc) were also performed. (**C**) For final reconfirmation (third round), 631 compounds prepared from re-sourced powders and were serially diluted (10 points, 1:3 dilution) and plated into Aurora 1536- well compound plates. Compounds (50 nl per well) were acoustically transferred into 1536-well assay plates. Multiple dose-respose assays such as *Pbluc*, *HepG2tox*, Ffluc, and ABS-Sybr were performed to determine IC_50_ in the third round of screening. A *P. vivax* liver schizont formation high-content assay in single-point (2X) was also performed.

The screen was run over an 18-month period, with ~20,000 compounds screened weekly (Fig. 1) to accommodate the time-consuming production of infected *Anopheles* mosquitoes. Each week, a team dissected ~1000 mosquitoes, from which the sporozoites were isolated and cleaned. Approximately 1000 sporozoites were added in a 5 ml volume per well plate by using a bottlevalve instrument to a lawn of HepG2-CD81 cells that had been prespotted into the 1536-well plates with 50 nl of compounds at 0.5 to 7.84µM(Fig. 1). After a 48-hour incubation, luciferin was added, and luminescence was measured. Because a compound that kills hepatocytes could result in reduced luciferase signal (because parasites presumably need a live hepatocyte for intracellular survival), we also tested a randomly selected subset of the compounds (~179,600) for hepatocyte toxicity using a Cell-Titer Glo assay (materials and methods) that measures the number of viable cells based on the amount of adenosine 5′-triphosphate (ATP) that is present (*HepG2tox*).

## Cheminformatic analysis of the primary screen

After collating the data and ranking the percentage inhibition for 538,273 compounds from the 1536-well screen, 64,172 compounds showed inhibition of >50%, and 21,336 showed inhibition levels of >75% (Fig. 1). Although a higher-thannormal number of false positives and negatives is expected for a single-point screen (data may be affected by edge effects, transfer errors, or variation in dispense volumes), the initial screen was informative. For example, the library included 197 ABS-active compounds that had been previously tested in a high-content imaging screen of *P. yoelii* liver stages (*9*), including 18 compounds with activity of less than 10 µM (data file S1). Of those, 15 showed >50% inhibition in the primary screen. Likewise, themajority of compounds that were considered inactive in the high-content imaging screen showed <75% inhibition here (129 out of 163) (data file S1).

**Fig. 2:**
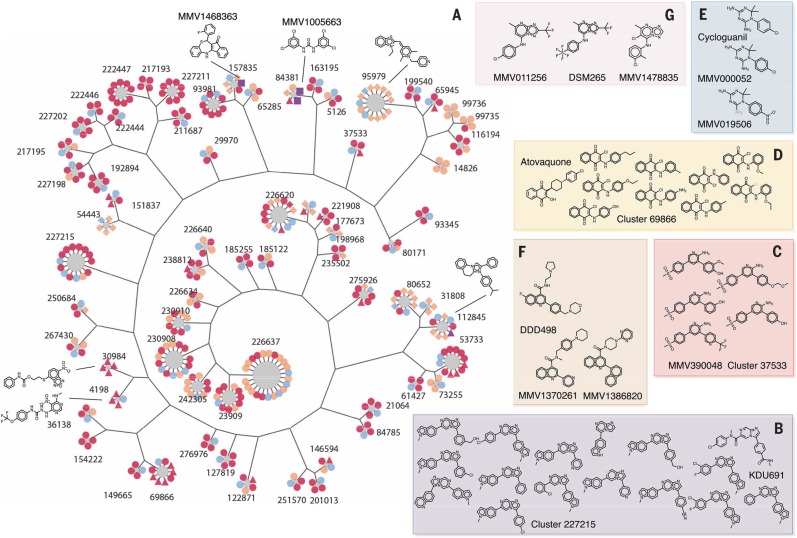
**Cluster analysis.** (**A**) For display, 405 compounds from 68 clusters that show a P value ≤ 0.05, cluster size ≥ 4, and hit fraction ≥ 0.75 are presented (data file S2). Most common substructure (MCS) per cluster is identified by using the top three active compounds, and a hierarchical tree was constructed from the MCSs to illustrate the intergroup connection. Compound members were then added to surround MCS nodes. All compound nodes are colored by hit status and shaped by other annotations. Primary hits are orange, reconfirmation hits (*Pbluc* IC_50_ < 10 µM) are red, third-round reconfirmation set (631 compounds) is purple, and others are light blue. P. falciparum asexual blood state–active compounds (*ABS-Sybr* IC_50_ < 10µM) are indicated by squares, luciferase inhibitors (*Ffluc* IC_50_ < *Pbluc* IC_50_ / 2) are indicated by triangles, hepatocyte toxic (primary *HepG2tox* > 50% or *HepG2tox* CC50 < *Pbluc* IC_50_ / 2) are indicated by diamonds, and the rest are shown as circles. (**B** to **G**) Active compounds in selected clusters [(B), (C), and (D)] with hit fractions of less than 0.75, as well as examples of singleton hits that are similar to the known antimalarial compounds, (E) cycloguanil, (F) DDD107498 (*13*), and (G) DSM265 (data file S3) (*16*).

To further evaluate the quality of the primary *Pbluc* data, we performed compound clustering (Fig. 2 and data file S2). We hierarchically clustered all 538,273 compounds in the primary screen and separated clusters using a minimum Tanimoto coefficient of 0.85 similarity (materials and methods). From this, we identified 281,090 compound clusters, with an average hit fraction of 0.016 ± 0.112. We calculated the probability of hit enrichment (>83% inhibition in *Pbluc*) by chance for each cluster (data file S2). Not unexpectedly, certain scaffold families with known activity against Plasmodium liver stages, such as the phosphatidylinositol-4-OH kinase (PI4K)– inhibiting imidazopyrazine family (*12*), included a high proportion of highly active compounds. For example, with an imidazopyrazine group (family 227215), 11 of the 14 members were highly active [average 81.1%, log10 probability of distribution by chance (log_10_ p) = –15.16] (Fig. 2A), and these were similar to the known imidazopyrazine KDU691 (*12*). Likewise, scaffold family 37533 consisted of four members where all four members in the library were more than 80% active (average 88.4%). All were very similar to MMV390048, another PI4K inhibitor (Fig. 2B). Atovaquone-like scaffolds (cluster 68966) were also present in the library and active at rates higher than expected by chance (eight of nine members active, log10 p = –10.88) (Fig. 2C). The hit fractions of cluster 69866, 37533, and 227215 (0.89, 0.75, and 0.79, respectively) are all in the top 1.1 percentile when considering the entire library.

Several additional scaffold families with known activity against malaria parasites were also found in the library and showed activity, although these families would not have been discovered with cluster analysis at the similarity thresholds used. For example, cycloguanil (83.4% inhibition) was found to be active in the 538,273-compound library but had only one close relative (> 85% Tanimoto similarity), which was not active (log_10_ p = –1.31). Relaxing the stringency, however, revealed three other relatives, one of which was also primary hit (log_10_ p = –2.42) (Fig. 2E). Likewise, compounds similar to DSM265 (*16*) and DDD107498 (Fig. 2, F and G) (*13*), two other potent compounds with nanomolar activity against exoerythrocytic stages, were also identified through relaxed similarity searching but were in clusters that might have arisen through chance (MMV011256, one of three active, log_10_ p = –1.35; MMV1478835, two of nine active, log10 p –1.40; MMV1370261, one of two active, log_10_p=–1.50) or were singletons (MMV1386820).

## Reconfirmation

To obtain a reasonable working set for reconfirmation (9989 compounds), we selected compounds that showed >83% *Pbluc* inhibition and *HepG2tox* of less than 50%, when available (data file S3). Cut off and filtering criteria did not bias physicochemical properties of primary hits, whose subset compounds had similar properties to the larger library, with weights of 388 Da (985.1 to 136.2), 1.1 hydrogen bond donors, 5.5 hydrogen bond acceptors, and 5.9 rotatable bonds on average (fig. S1 and table S2). To confirm primary hits, the location of wells with possible active compounds were identified, and then a hit-picking instrument was used to reassemble a collection for reconfirmation (data file S3). We arrayed these hit compounds in eight-point dose-response format (5 nM to 10 µM) and reassessed their potency and efficacy in duplicate in three different assays—including the Pbluc assay, the *HepG2tox* assay, and a counterscreen— to test whether a given compound would inhibit firefly luciferase in a biochemical assay (*Ffluc*). Altogether, complete *Pbluc* dose-response curves could be fitted for 5942 compounds [halfmaximal inhibitory concentration (IC_50_) < 15.4µM, average 4.71 µM], and 681 showed complete *Pbluc* inhibition at all tested concentrations (18 compounds) or an IC_50_ of <1 µM (663 compounds) (data file S3). However, of the 5942 reconfirmed hits, 790 demonstrated moderate inhibition of hepatocyte viability [*HepG2tox* CC50 (50% cytotoxicity concentration) < 2X *Pbluc* IC_50_ and *HepG2tox* CC_5_0 < maximum tested concentration], and 465 of these also interferedwith firefly luciferase production (*Ffluc* IC_50_ < 2X *Pbluc* IC_50_ and *Ffluc* IC_50_ <maximum tested concentration) (data file S3).

Adding these data to our cluster analysis (Fig. 2) showed enrichment of false positives arising from the use of luciferase as a surrogate for parasite development. For example, 526 scaffolds of the imidazopyridine-carboxamide group (128188) were found in the library of 538,273 compounds, of which 145 were in the hit list (log_10_ p < –100) (data file S2). Of these, 116 showed biochemical luciferase inhibition <10 µM (29 less than 1 µM) (data files S2 and S3). Visualization showed that other less abundant scaffolds, such as those in cluster 30984 or 4198, also showed enrichment of luciferase activity at higher rates than expected by chance (Fig. 2 and data file S2). Likewise, some enriched scaffold families (log_10_ p = –14.45) were clearly toxic to human cells (cluster 95979) (Fig. 2 and data file S2).

To further establish the integrity of the hits, we next assembled a collection of compounds for third-round, independent testing. The nontoxic second round hits were filtered through the general medicinal chemistry compound filters by using Biovia ScienceCloud. This filtering removed 619 compounds that either failed Lipinski’s rule of 5 (based on calculated properties) as a surrogate for “drug-like”; had structural features consistent with high chemical reactivity or instability; had a high likelihood of nonspecific covalent interaction, such as thiourea; or was an enone, which would react with nucleophiles such as thiols or disrupt disulphide bonds (*17*). The remaining set was then prioritized on the basis of potency (IC_50_) and calculated lipophilicity (*cLogP*), yielding 953 compounds prioritized as follows: 445 actives with IC_50_ < 1 µM (most potent—no additional cLogP filter), 268 actives >1 µM but clogP < 2.5 (low lipophilicity compounds with lower potency), and 240 actives >1 µM but 2.5 < clogP < 3 (as above, with moderate lipophilicity).

With this set of 953 compounds, we interrogated the databases of commercially available compounds for acquisition, confirmation, and further profiling; 631 (repurchased validation set) were available and thus further characterized (data file S4). These compounds were arrayed within the 1536-well plate format into 10-point dose response, tested in duplicate (technical replicates), and counterscreened in multiple different assays, repeated up to four times with different sporozoite batches. Two separate *HepG2tox* assays and one *Ffluc* also were performed. Retesting showed a high reconfirmation rate (82%with an average *Pbluc* IC_50_ < 10 µM) and 376 compounds with *Ffluc* IC_50_ and *HepG2tox* CC50 < 2× *Pbluc* IC_50_ (fig. S2). All compounds were also tested in dose-response format against ABS of the multidrug-resistant *P. falciparum* Dd2 strain, using a standard SYBR green incorporation bloodstage assay that identifies compounds that prevent growth and development (*ABS-Sybr*; four biological replicates) (*6*).

## Activity against *P. vivax*

To better understand species specificity, the aforementioned repurchased validation set (631), reconfirmed in eight-point dose assays with *Pbluc*, was also tested against the human pathogen *P. vivax.* For this, we used a single-point high-content imaging screen (*PvHCI*), with primary human hepatocytesmaintained in 384 well plates.Hepatocytes were inoculated with *P. vivax* sporozoites dissected frommosquitoes fed with blood obtained from malaria patients. The following day, compounds were added to a final concentration of 10 µM and then reapplied at days 2 and 3 and 4. After compound treatment, cultures were fixed on day 6 after infection, stained with immunoreagents against PvUIS4 (materials and methods), and we performed high-content imaging and analysis to score eachwell for growth of liver schizonts. Of the 631 compounds screened in this single-point assay, 163 were confirmed to inhibit *P. vivax* schizont growth by at least 70% (normalized to positive control KDU691) (data file S4) in at least one of two biological replicates, and 91 were confirmed in both replicates (fig. S2). This confirmation rate was achieved despite several critical differences between the rodent and human assays, including hepatic metabolism of unoptimized compounds and addition of compound on day 1 after infection (*PvHCI*) instead of preinfection (*Pbluc*). Furthermore, 12 compounds (fig. S2) were considered not reconfirmed in a third round of *P. berghei* testing but were nevertheless still active in *P. vivax* (for example, MMV1288041 inhibited *P. vivax* by 100% in both replicates and had an IC_50_ of *P. berghei* 3.27 µM in second-round testing), highlighting how experimental design and analysis constraints (for example, curve-fitting compounds that did not fully inhibit growth in *Pbluc* at 10 µM, the highest concentration tested, would be considered “not confirmed”) could lead to possible false negatives. On the other hand, compounds could lose potency over repeated use owing to freeze-thaw and hydration, there could be differences in liver-stage schizogony between *P. vivax* and *P. berghei* (liver schizonts produce far greater quantities of liver merozoites over six additional days of development in comparison to the 72-hour liver cycle of *P. berghei*), and the wild-type *P. vivax* may be more drugsensitive than *P. berghei*–ANKA-GFP-Luc-SMCON or *P. falciparum* Dd2, both of which are resistant to some antifolates.

## Activity against P. falciparum blood stages

To further study the function of the third-round compounds, we next tested whether the repurchased validation set of 631 would be active against *P. falciparum* ABS parasites. The active set showed several known chemotypes. One example, MMV1468363, showed a high degree of similarity to tetracyclic benzothiazepines, which are known cytochrome bc1 inhibitors (*18*). There were seven closely related members of the tetracyclic benzothiazepines scaffold family in the larger library, and six of the seven showed inhibition rates of greater than 85% in the primary screen (log_10_ p = –7.969, cluster 157835) (Fig. 2). Another recognizable scaffold was that of MMV1005663, similar to the possible FabI inhibitor trichlocarban, as well as the liver stage–active malaria box member, MMV665852 (*19*). This latter compound is associated with amplifications of the gene encoding phospholipid flippase, *pfatp2* (*19*). This compound series (cluster 84381) was represented multiple times in the library, with all five tested members showing strong inhibition (average 93%, log_10_ p = –7.34) in the primary screen (Fig. 2).

Three hundred ninety-eight liver-active compounds were inactive in *P. falciparum* ABS-Sybr (average IC_50_ of > 10 µM) (fig. S2), highlighting the rich new untapped chemical space that was sampled by using this screening cascade when compared with the ABS-first cascade. Although some could be due to species-specific activities, 130 compounds were active against both *P. vivax* (in at least one replicate) and *P. berghei* schizonts (fig. S2) but were inactive against ABS. Although our use of antifolate-resistant *P. berghei* and a multidrug-resistant *P. falciparum* strain may influence this result, most of these scaffolds likely affect parasite or human processes that exist only in the hepatic stages. These scaffolds generally did not show any similarities to known antimalarials, but several of these were supported by strong statistical overrepresentation in the primary screen (Fig. 2 and data file S2). For example, seven of theninemembers of the imidazoquinoline cluster (112845) were active in the primary screen (log_10_p= –8.89) (Fig. 2).

## Mechanism of action studies reveal an abundance of mitochondrial transport chain inhibitors

**Fig. 3:**
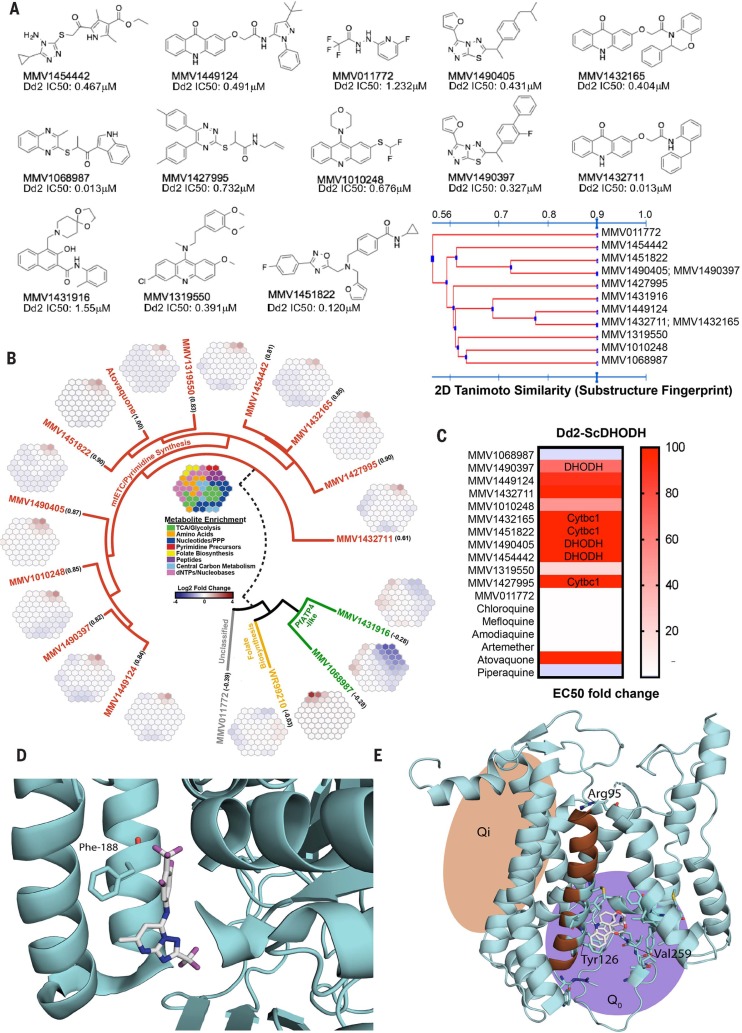
**Target identification studies.** (**A**) Chemical structures and IC_50_ of select antimalarial compounds identified as hits. Tanimoto clustering demonstrates that most molecules are structurally distinct, although some share similar scaffolds. (**B**) Metabolomic analysis reveals that 10 of the 13 compounds likely target the mETC and pyrimidine biosynthesis pathways. Robust increases in pyrimidine biosynthesis precursors N-carbamoyl-Laspartate (CA) and dihydroorotate (DHO) are signatures of metabolic disruption of de novo pyrimidine biosynthesis. The metaprints for MMV1068987 and MMV1431916 are similar to the metaprint of the PfATP4 inhibitor KAE609, whereas the metaprint for MMV011772 is inconclusive. The numbers below each compound name indicate the Pearson correlation with an atovaquone profile (fig. S3). (**C**) IC_50_ of each compound in Dd2 cells expressing S. cerevisiae DHODH normalized to parent. The transgenic Dd2-ScDHODH strain expresses the cytosolic type 1 DHODH from S. cerevisiae (ScDHODH) and is resistant to P. falciparum mETC inhibitors. Ablation of compound activity in this cell line relative to its parent indicates inhibition of DHODH or downstream effectors in the mETC such as Cytbc1. Atovaquone, a known Cytb inhibitor, was included as a positive control, whereas other licensed antimalarials with mETC-independent mechanisms of action serve as negative controls. (**D**) Location of Phe^188^Ile mutation found in whole-genome sequences of MMV1454442-resistant parasites by using a crystal structure of PfDHODH (4ORM) (27). Amino acid residue 188 is highlighted in magenta.The structure shows a known PfDHODH inhibitor, DSM338 (27), cocrystalized with the protein. (**E**) Homology model of PfCytb (from PDB 1BE3) (35) with Tyr^126^Cys and Val**^259^**Leu mutations (highlighted in magenta) from MMV1432711-resistant parasites.The Arg**^95^** has previously been implicated in atovaquone binding and resistance (36).

To further investigate the mechanism of action of 13 of the most potent ABS hits (Fig. 3A), we acquired additional compound from commercial vendors. Because most, if not all, target identification methods require activity in *P. falciparum* blood stages, all compounds selected for initial target discoverywere active against ABS parasites, with an average IC_50_ of 465 nM(range of 13 nMto 1.2 µM).

As an initial pass, we first subjected the compounds to metabolic profiling (*20*). This liquid chromatography–mass spectrometry (LC-MS)– based method measures several hundred metabolites and identifies those that show statistically significant increases or decreases upon parasite compound exposure (data file S5).Whereas three gave ambiguous results, 10 of the 13 analyzed scaffolds gave a metabolic profile signature analogous to that of atovaquone (fig. S3), indicating that these 10 most likely interfere with one or more targets in the mitochondrial electron transport chain (mETC), a known druggable pathway for P. falciparum blood and liver stages (Fig. 3A). The set of 13 compounds represents 11 distinct scaffolds (Fig. 3B), so this degree of functional overlap would not have been predicted by structure alone. To our knowledge, none of the molecules have been previously identified as acting against the mitochondrial electron transport pathway.

To further confirm that these 10 compounds (representing eight chemotypes) inhibited the mETC, we took advantage of a transgenic parasite line that overexpresses the Saccharomyces cerevisisae dihydroorotate dehydrogenase (Dd2- ScDHODH) (*21*). Unlike the type-2 P. falciparum enzyme that is dependent on cytochrome bc1 for ubiquinone, the cytosolic type-1 yeast enzyme can use fumarate as an electron acceptor. This allows the transgenic parasites to bypass the need for ETC activity to provide ubiquinone to PfDHODH (*21*). Compounds that target PfDHODH or other enzymes along the mETC lose potency in the Dd2-ScDHODH transgenic cell line. As expected, the Dd2-ScDHODH parasites showmarked (24.8 to >1000-fold) resistance to the 10 compounds with the mETC metabolic profile (Fig. 3C and table S3). Furthermore, a variation of this functional assay can distinguish between inhibitors of PfDHODH and cytochrome bc1. Specifically, the addition of proguanil to Dd2-ScDHODH parasites restores the inhibitory capabilities of cytochrome bc1 inhibitors; however, growth is not affected in the case of PfDHODHinhibitors. Three out of six mitochondrial inhibitors tested in these conditions were not inactivated by proguanil, suggesting a profile consistent with PfDHODH inhibition (Fig. 3C and table S4). To further investigate mitochondrial inhibition, and because there are multiple potential targets, we used an in vitro evolution and whole-genome analysis (IViEWGA) approach (*22*) to further elucidate the molecular target of several of the compounds, including MMV1454442, MMV1432711, and MMV142795. First, three independent lines resistant to MMV1454442 were isolated after growing in sublethal concentrations of compound. The resistant clones showed an average 4.2-fold shift in the IC_50_ (range of 1.9 to 9.4) (table S5). Whole-genome sequencing of the nine clones (three each from three independent selections), as well as the drug-sensitive parent clone to 78-fold coverage (table S6), revealed that the resistant lines carried either a single-nucleotide variant Phe188Ile (Fig. 3D and data file S6) or a copy number variant (table S8) in P. falciparum dihydroorotate dehydrogenase (PF3D7_0603300), which is a well-validated drug target in P. falciparum (*16*). This result is consistent with proguanil not affecting growth inhibition in Dd2-ScDHODH parasites (Fig. 3C).MMV1454442, an amino-triazol, although somewhat similar to a pyrrole-based DHODH inhibitor (*23*), is a previously unidentified chemotype, which would not have been predicted with structural information alone. The Phe188 residue is located in the species-selective inhibitor-binding pocket of PfDHODH (*24*) and has been shown to be in contact with the known DHODH inhibitor leflunomide (*25*) and the triazolopyrimidine DSM338 (Fig. 3D) (*26*). Furthermore, mutation of this residue has been shown to confer resistance to the alkylthiophene inhibitor Genz-669178 (*27*), suggesting that MMV1454442 likely shares the same space (Fig. 3D).

IViEWGA of MMV1432711-resistant parasites revealed they had acquired one of two nonsynonymous single-nucleotide variants (SNVs) in the gene encoding cytochrome b (data file S7). The amino acid mutations found, Tyr^126^Cys and Val^259^Leu, are located within helix C in the ubiquitinol-binding pocket of cytochrome b, a catalytically important subunit of the cytochrome bc1 complex that contains two reaction sites, Q0 (ubiquitinone reduction site) and Qi (ubiquitinol oxidation site). MMV1432711 has a chemical scaffold similar to that of the Qi inhibitors, sowe used a homology model of PfCYTb (Fig. 3E) to resolve the mode of binding. Docking into the model showed that MMV1432711 is likely a class II inhibitor. The allele Y126C was previously reported to confer resistance to decoquinate (*28*) and MMV008149 (*29*). To our knowledge, allele Val^259^Leu has not been reported in the literature.

For compound MMV1427995 {2-[(5,6-diphenyl- 1,2,4-triazin-3-yl)thio]-1-(pyrrolidin-1-yl)propan-1- one}, in vitro evolution studies yielded two resistant lines that were cloned for further phenotyping and whole-genome sequencing (tables S5 and S6). Clones showed an average 2.6-fold IC_50_ shift (range of 1.8- to 3.4-fold) in susceptibility to MMV1427995 (table S5). Sequencing revealed that both clones carried the amino acid mutation, Arg^95^Lys, in cytochrome b, located in the matrixoriented region of the protein after the second transmembrane domain (Fig. 3E and data file S6). One clone also carried an additional Pro^102^Thr mutation in cytochrome c oxidase subunit 1. This mutation is located between the second and third transmembrane domain, is not located in any of the iron or copper redox centers, and is, to our knowledge, the first described mutation in cytochrome c oxidase selected for during compound exposure. However, this mutation did not induce a higher resistance level than did the Arg^95^Lysmutation alone andmay thus represent a compensatory mutation. MMV1427995 is a different scaffold family (also overrepresented in the initial set of screening hits) from known *P. falciparum cytochrome* bc1 inhibitors, and its target would not have been predicted through similarity searching.

Given the high number of mitochondrial inhibitors in the dataset, we further examined the set of 631 compounds (repurchased validation set). All 631 compounds were tested in duplicate in eight-point ABS dose response in two *P. falciparum* D10–derived lines (*21*), one of which expresses ScDHODH (fig. S4 and data file S7) in the presence and absence of 1 mM proguanil in duplicate (~80,000 data points). Of the 136 compounds with ABS activity, visual inspection showed that 78 were likely not mitochondrial inhibitors, and 58 showed profiles consistent with mitochondrial inhibition (figs. S4 and S5 and data file S7). Of these, 10 were clear DHODH inhibitors (including the three shown in Fig. 3), one was a potential DHODH inhibitor, and 47 were likely or possible cytochrome bc1 inhibitors (including all nine from Fig. 3 that were tested). Strong nonrandom structure-activity relationships are evident (fig. S5), validating the assay. For example, six of the seven compounds that were more than 55% similar to MMV1042937 (fig. S5, fourth row) in the set of 631 were predicted to be cytochrome bc1 inhibitors (log_10_ p = –6.09). The seventh, MMV1457596, was missed because the IC_50_ were all >10 µM, but visual inspection of the curves showed an ~75% reduction in signal at 10 µM for the ScDHODH line relative to the PfDHODH line (data file S7).

## Discussion

Previous open-access high-throughput screens have had a great impact on malaria research, and we anticipate that our data provide a rich resource in the search for new antimalarials. Despite the reliance on a nonhuman malaria species for screening, the repeated rediscovery of chemotypes with known, potent activity against *P. falciparum* blood stages and *P. vivax* liver schizonts, or clinical efficacy in humans, shows that the data from this rodent malaria model are predictive. Furthermore, liver schizonticidal activity is a required component of next-generation antimalarials proposed byMedicines forMalaria Venture, critical components of which include prophylactic and chemoprotective liver-stage efficacy after single exposure (TCP4) (*30*). Another advantage of the *Pbluc* assay is the reduced metabolic capacity of the host cells used; this should result in a higher hit rate with metabolically liable compounds in a high-throughput phenotypic screen. Last, although it is possible that some compounds were missed through the use of a rodent parasite, rodent malaria remains an efficient and important in vivo model for drug efficacy testing; compounds that do not act in this model would be costlier to progress into animal causal prophylaxis testing. In addition, some of the compounds that have activity here may eventually show activity against *P. vivax* hypnozoites, and if a radical cure chemotype is to be found, its discovery would be made much more likely through elimination of liver schizontinactive compounds. The use of the low-cost rodent model allowed a much higher number of compounds to be evaluated than would be possible with human parasites, which can be difficult and expensive to acquire. The large size of the library used here facilitated compound clustering and allowed a probabilistic identification of active families.

The high number of parasite mitochondrial inhibitors (57) that were discovered with combined target identification methods is perhaps not surprising given that compounds were selected for target identification on the basis of potency, and substantial work has shown that mitochondrial inhibitors are very potent antimalarials. Nevertheless, the dataset contains a rich set of other ABS scaffolds that could still be good starting points for medicinal chemistry efforts designed to improve potency, selectivity, and reduced toxicity. The picomolar inhibitor and clinical candidate KAE609 was derived from an ~90 nM IC_50_ starting point that was discovered in a high-throughput ABS screen (*31*).

Although our target identification efforts showed that several compounds with activity across species were mostly in known target classes, a much larger number of compounds were active in the liver stages and had no activity in the blood stages. These presumably act against host pathways that the parasite requires for development, or alternatively, the infected cell may be weakened by the presence of a parasite and be more susceptible to killing by compounds that target essential host cellular processes. It is worthwhile to mention that because there are no reported antimalarial compounds with exclusive prophylactic activity, and methods for target identification are not readily available for compounds that do not act in the blood stage, we are unable to conclude much about their mechanism of action. Nevertheless, the scaffolds should still represent important starting points for prophylaxis stage drugs and exploring new mechanism of actions.

In theory, liver-stage antimalarial compounds could function as more cost-effective “chemical vaccines” that could replace conventional vaccines inmalaria-elimination campaigns, provided sufficient safety. A fully protective conventional vaccine has never been developed for malaria (*32*); vaccines are very species-specific (and potentially strain-specific), whereas chemotherapy is generally not, and vaccines, which typically consist of recombinant protein or a heat-killed organism, require maintenance of a cold chain. In the case of the irradiated or attenuated cryopreserved sporozoite vaccine (*33*), the cost of goods (sporozoites that are hand-dissected from infected mosquitoes) will be much higher than a small-molecule therapy. We estimate that it costs 10 cents per well to create the 1000 sporozoites needed for one well of a 1536-well plate. Creating enough sporozoites to immunize a humanwould cost many times this amount.

In an analogous situation, long-acting, injectable HIV drugs have now been developed, and their deploymentmay help to prevent the spread of the HIV virus (*34*). Such intramuscular or subcutaneous chemoprotection injections or “chemical vaccines” can be deployed in resource-poor settings, and patient compliance is not as much of an issue as with standard, orally available tablet drugs. Because the injectable typically consists of a small molecule, refrigeration and maintenance of a cold chain may not be needed. The cost of goods for an injectable that needs to be supplied once every 1 to 3 months will most likely be lower than the cumulative cost of a tablet that needs to be taken every day, even considering the cost of a syringe and a health care worker to provide delivery. There are also other low-cost deliverymethods, such as patches. Thus, it seems entirely feasible that a contribution to the eradication of malaria could come through the use of a long-acting chemical vaccine.

## Materials and methods

A detailed description is provided in the supplementary materials.

## Supplementary Material

Open-source discovery of chemical leads for next-generation chemoprotective antimalarials

Open-source discovery of chemical leads for next-generation chemoprotective antimalarials

Open-source discovery of chemical leads for next-generation chemoprotective antimalarials

Open-source discovery of chemical leads for next-generation chemoprotective antimalarials

Open-source discovery of chemical leads for next-generation chemoprotective antimalarials

Open-source discovery of chemical leads for next-generation chemoprotective antimalarials

Open-source discovery of chemical leads for next-generation chemoprotective antimalarials

Open-source discovery of chemical leads for next-generation chemoprotective antimalarials

## Data and materials availability

DNA sequences have been deposited in the shortread sequence archive (www.ncbi.nlm.nih.gov/sra) under accession no. SRP134381. Metabolomics data has been deposited in the NIH Metabolomics Workbench under accession no. PR000719. All other data are available in the manuscript or the supplementary materials. This work is licensed under a Creative Commons Attribution 4.0 International (CC BY 4.0) license, which permits unrestricted use, distribution, and reproduction in any medium, provided the original work is properly cited. To view a copy of this license, visit http://creativecommons.org/licenses/by/4.0/. This license does not apply to figures/ photos/artwork or other content included in the article that is credited to a third party; obtain authorization from the rights holder before using such material.

## SUPPLEMENTARY MATERIALS

www.sciencemag.org/content/362/6419/eaat9446/suppl/DC1

Tables S1 to S8

References (*37–46*)
