# Supplementary material for: Open-source discovery of chemical leads for next-generation chemoprotective antimalarials
Source: Science. 2018 Dec 7;362(6419):eaat9446. doi: 10.1126/science.aat9446 (PMC6516198; doi:10.1126/science.aat9446)
Supplement: Open-source discovery of chemical leads for next-generation chemoprotective antimalarials [file Science-362-eaat9446-s001.pdf]

www.sciencemag.org/content/362/6419/eaat9446/suppl/DC1

Supplementary Material for

Open-source discovery of chemical leads for next-generation chemoprotective antimalarials

Yevgeniya Antonova-Koch, Stephan Meister, Matthew Abraham, Madeline R. Luth, Sabine Ottilie, Amanda K. Lukens, Tomoyo Sakata-Kato, Manu Vanaerschot, Edward Owen, Juan Carlos Jado Rodriguez, Steven P. Maher, Jaeson Calla, David Plouffe Yang Zhong, Kaisheng Chen, Victor Chaumeau, Amy J. Conway, Case W. McNamara, Maureen Ibanez, Kerstin Gagaring, Fernando Neria Serrano, Korina Eribez, Cullin McLean Taggard, Andrea L. Cheung, Christie Lincoln, Biniam Ambachew, Melanie Rouillier, Dionicio Siegel, François Nosten, Dennis E. Kyle, Francisco-Javier Gamo, Yingyao Zhou, Manuel Llinás, David A. Fidock, Dyann F. Wirth, Jeremy Burrows, Brice Campo, Elizabeth A. Winzeler*

*Corresponding author. E-mail: ewinzeler@ucsd.edu

Published 7 December 2018, *Science* **362**, eaat9446 (2017)
DOI: 10.1126/science.aat9446

This PDF file includes:

Materials and Methods
Figs. S1 to S4
Tables S1 to S8
References

Other Supplementary Material for this manuscript includes the following:
(available at www.sciencemag.org/content/362/6419/eaat9446/suppl/DC1)

Data Files S1 to S7 as separate Excel files

Table of Contents

MATERIALS AND METHODS 3
FIGURE S1 11
FIGURE S2 12
FIGURE S3 13
FIGURE S4 14
FIGURE S5 15
TABLE S1 16
TABLE S2 17
TABLE S3 18
TABLE S4 19
TABLE S5 20
TABLE S6 21
TABLE S7 22
TABLE S8 23
DATA S1. (SEPARATE FILE) 24
DATA S2. (SEPARATE FILE) 24
DATA S3. (SEPARATE FILE) 24
DATA S4. (SEPARATE FILE) 24
DATA S5. (SEPARATE FILE) 24
DATA S6. (SEPARATE FILE) 24
DATA S7. (SEPARATE FILE) 24

Materials and Methods

Compound Libraries

The compound library was obtained from Charles River. The library consists of 538,273 compounds in DMSO at concentrations ranging from 0.5 to 7.84mM (average 2mM). Compounds were screened at 0.250µM to 39µM (average 10µM). Library characteristics are listed in Table S1.

Compound Suppliers

Compounds that were further evaluated for metabolomic studies and DHODH inhibition were purchased from the following supplier: MMV1068987 (MolPort-009-487-577), MMV1490397 (MolPort-005-913-878), MMV1449124 (MolPort-005-706-859), MMV1432711 (MolPort-005-733-379), MMV1010248 (MolPort-001-739-182), MMV1432165 (MolPort-005-733-376), MMV1451822 (MolPort-007-324-958), MMV1490405 (MolPort-000-844-049), MMV1454442 (MolPort-004-259-328), MMV1319550 (MolPort-001-739-194), MMV1427995 (MolPort-009-388-424), MMV011772 (MolPort-002-872-109), MMV1431916 (MolPort-009-260-425). Compound purity was assessed by mass-spectrometry and is shown in Table S8.

Plasmodium berghei exoerythrocytic parasites

Anopheles stephensi mosquitoes infected with *P. berghei*-ANKA-GFP-Luc-SMCON (Pb-Luc)(*15*) sporozoites bearing the antifolate-resistance-conferring *Toxoplasma gondii* dihydrofolate reductase-thymidylate synthase selectable marker, pyR2(*15*) and which is integrated into the *d-ssu-rrna* fragment that serves as a target for integration into the genome in the nonessential *c-* or *d-ribosomal rna* gene unit(*36*). The GFP-Luc reporter is driven by the constitutively active *pbeef1aa* promoter (*15*). These were received from the Insectary Core Facility at New York University. The Pb-Luc sporozoites were obtained by dissection of infected *Anopheles stephensi* mosquito salivary glands. Dissected salivary glands were homogenized in DMEM media (Life Technology, CA) using a glass tissue grinder, filtered twice through a 20 µM nylon net filter (Steriflip, Millipore), and counted using a Neubauer hemocytometer (C-Chip, InCyto, Republic of Korea). The sporozoites were kept on ice until needed.

Culturing asexual erythrocytic-stage *Plasmodium falciparum* parasites

P. falciparum Dd2 strain was cultured using human erythrocytes obtained from healthy adult donors (196 female, 247 male over the course of the study) enrolled in The Scripps Research Institute, Normal Blood Donor Service Program (IRB-12-5933), complete medium (RPMI 1640 with l-glutamine, without phenol red (Life Technologies, CA) supplemented with 4.3% heat-inactivated O^+ human serum, 0.2% AlbuMAX II lipid-rich BSA, 0.014mg/mL hypoxanthine, 3.4mM NaOH, 38.4mM Hepes, 0.2% glucose, 0.2% sodium bicarbonate, and 0.05mg/mL gentamicin) containing 5% hematocrit in a low-oxygen atmosphere (1% oxygen, 3% carbon dioxide, and 96% nitrogen) at 37°C until parasitemia reached 3-8%. Parasitemia was determined by visual inspection of blood smears stained with Giemsa.

The Dd2-attB and Dd2-ScDHODH parasite cell lines have been described previously(*18*) and were maintained under the same culture conditions as described above for wild-type Dd2 parasites.

Cell lines

Human hepatoma cells, HepG2-A16-CD81EGFP(*37*), stably transformed to express a GFP-tetraspanin receptor CD81 fusion protein, were cultured at 37°C and 5% CO_2 in DMEM (Life Technology, CA) supplemented with 10% FBS, 0.29mg/mL glutamine, 100 units of penicillin, and 100μg/mL streptomycin. Assay media (DMEM without phenol Red (Life Technology, CA) supplemented with 5% FBS, 1.45mg/mL glutamine, 500 units of penicillin, and 500μg/mL streptomycin) was used for the *Pbluc* and *HepG2tox* assays.

Luciferase-based high throughput screening: *P. berghei*-Luciferase liver stage assay (*Pbluc*) and HepG2 cytotoxicity assay (*HepG2tox*)

For the *Pbluc* high-throughput screen, we used *P. berghei* because of its higher infection rates in immortal human hepatocyte cell lines, making it more conducive to high-throughput screening than the human malaria parasites. HepG2-A16-CD81EGFP(*37*) cells were cultured at 37°C in 5% CO_2 in DMEM media as described in Cell Lines section above. For both *Pbluc* and *HepG2tox* assays, 20-26 hour prior to sporozoites infection, $3x10^3$ of the HepG2-A16-CD81EGFP20 cells in 5 μl of assay medium (DMEM without Phenol Red (Life Technologies, CA), 5% FBS, and 5x Pen Strep Glutamine (Life Technologies, CA)) at concentration $6x10^5$ cells/ml were seeded in white solid bottom 1536-well plates (custom GNF mold ref# 789173-F, Greiner Bio-One). For primary single dose screens, 18 hours prior to infection, 50nL of compound in DMSO (0.5% final DMSO concentration per well) was transferred with a PinTool (GNF Systems) into the assay plates. For IC_{50} determinations in second and third round of the screenings, 18 hours prior to infection 50nL of compounds in 1:3 serial dilutions in DMSO (0.5% final DMSO concentration per well) were transferred with either Echo Liquid Handler (Labcyte) (for the second round of screening) or Acoustic Transfer System (ATS) (Biosero) (for the third round of screening). Atovaquone (5μM) and puromycin (10μM) at a single concentration were used as positive controls for the *Pbluc* and *HepG2tox*, respectively. 0.5% DMSO was used as negative control for both assays. *Pbluc* sporozoites were freshly dissected from the infected *A. stephensi* salivary glands, filtered twice through a 20μm nylon net filter (Steriflip, Millipore), counted in a hemocytometer, and adjusted to final concentration 200 sporozoites per 1μl in the assay media (DMEM without Phenol Red (Life Technologies, CA), 5% FBS, and 5x Pen Strep Glutamine (Life Technologies, CA). To infect the HepG2-A16-CD81EGFP cells, $1x10^3$ sporozoites per well (5μl) were added with a single tip Bottle Valve liquid handler (GNF), and the plates were spun down at 37°C for 3 minutes with a centrifugal force of 330xg on normal acceleration and brake setting (Eppendorf 5810 R centrifuge). The HepG2-A16-CD81EGFP cell designated for toxicity studies were left uninfected, with 5μl of additional assay media added to each well to maintain equal concentrations of compounds with *Pbluc* infected plates. The plates were then incubated at 37°C for 48 h in 5% CO_2 with high humidity to minimize media evaporation and edge effects.

Bioluminescence quantification for *Pbluc* and *HepG2tox* assays

After incubation at 37°C for 48 h, the EEF growth and HepG2-A16-CD81EGFP cells viability were assessed by a bioluminescence measurement as follows: Media was removed by spinning

the inverted plates at 150xg for 30 seconds; 2µl per well of BrightGlo (Promega) for quantification of *Pbluc* EEFs or CellTiterGlo (Promega) reagent (diluted 1:2 with deionized water) for quantification of HepG2-A16-CD81EGFP cell viability (*HepG2tox*) were dispensed with the MicroFlo (BioTek) liquid handler. Immediately after addition of the luminescence reagent, the luminescence was measured by the Envision Multilabel Reader (PerkinElmer).

<u>Hit analysis and IC_{50} determination for *Pbluc* inhibition and *HepG2tox* assays</u>

For the first round of screening for 538,273 compounds in single point concentration in the 1536-well plate, the lack of inhibition (baseline) was defined as the average bioluminescence of 64 wells with 0.5% DMSO, and the background inhibition was defined as average luminescence of 64 wells with 10µM atovaquone. The *Pbluc* growth inhibition was determined as a ratio of luminescence reads from every well with a compound minus the background inhibition readings with this difference divided by the baseline minus background inhibition readings. For the second round of screening first round of reconfirmation for 10,000 compounds in 8 points (1:3) dilution series, the background for the EEF inhibition was defined as the average of 16 wells with atovaquone at concentration of 10µM, and the background for the *HepG2tox* was defined as the average of 16 wells with puromycin at concentration of 10µM. IC_{50} values were determined using the average normalized bioluminescence intensity of 2 wells per concentration from 2 technical replicates of the 1536-well plates, and a nonlinear variable slope four-parameter regression curve fitting model in Prism 6 (GraphPad Software Inc.).

For the second reconfirmation round of screening (third round of screening for 631 compounds resourced from powder), the background for the *Pbluc* inhibition was defined as average bioluminescence of the 16 wells with atovaquone at single concentration of 5µM, and the background for the *HepG2tox* was determined as the average of the 12 wells with puromycin at single concentrations of 5µM. The baseline was defined as average bioluminescence of the 150 well with 0.5% DMSO. The data was normalized against positive and negative controls and IC_{50} were determined with Levenberg-Marquardt algorithm for curve fitting of the dose response data (fit parameters as min = 0.0, max ≥ 80.0, slope ≥ 0.0) using CDD Vault from Collaborative Drug Discovery (Burlingame, CA. www.collaborativedrug.com) All data in this study were archived using the CDD Vault from Collaborative Drug Discovery (Burlingame, CA, www.collaborativedrug.com).

<u>Recombinant *Photinis pyralis* luciferase (*Ffluc*) inhibition assay</u>

The luciferase inhibition assay was performed for the hits of the primary screen in the first and second rounds of reconfirmation (second and third rounds of screening). In the 1st round of reconfirmations (second round of screening), 50 nL of compounds in 1:3 serial dilutions in DMSO (0.5% final DMSO concentration per well) was transferred with an Echo Liquid Handler (Labcyte) into white, solid-bottom 1536-well plates (custom GNF mold ref# 789173-F, Greiner Bio-One). Next, 8µl of the Quantilum Luciferase (catalog #E1701) in 1:1000 dilution in DMEM (Life Technologies, CA) was dispensed with the MicroFlo (BioTek) liquid handler. As positive control 100nL resveratrol (Sigma catalog #5010) was added to each well to a final concentration of 500µM. Plates were incubated at 37°C for 3 hours. After the incubation 2µL of BrightGlo (Promega) was added to the wells with the MicroFlo liquid handler (BioTek). Immediately after addition of Bright Glo (Promega), the plates were read by an EnVision Multilabel reader (PerkinElmer). In the third round of screening (second round of reconfirmations for the 631

compounds resourced from powder), 40nL of compounds in 1:3 serial dilutions in DMSO (0.5% final DMSO concentration per well) were transferred in white solid bottom 1536-well plates (custom GNF mold ref# 789173-F, Greiner Bio-One) with Acoustic Transfer System (ATS) (Biosero). 8 µl of 20 pM QuantiLum Recombinant Luciferase (Promega, catalog #E1701A) dilution in assay medium (DMEM without Phenol Red (Life Technologies, CA), 5% FBS, and 5x Pen Strep Glutamine (Life Technologies, CA)) were dispensed with the MicroFlo (BioTek) liquid handler. Luciferase Inhibitor-I (non-competitive) (Calbiochem, Ref # 119113) and Luciferase Inhibitor-II (competitive) (Calbiochem, Ref # 119114) at single concentration of 10µM and 9.8µM respectively were used as positive controls. As a negative control 0.5% DMSO was used. The plates containing tested compounds and QuantiLum Luciferase were incubated at RT for 15 min. After the incubation, 2µL of BrightGlo (Promega) was added to the wells with the MicroFlo liquid handler (BioTek). Immediately after addition, the plates were read by an EnVision Multilabel reader (PerkinElmer).

IC_{50} determination for *Ffluc*

To determine IC_{50} for luciferase inhibition in the luciferase inhibition assay in the first round of reconfirmations (second round of screening), the background luminescence was defined as the average of 48 wells with 500µM resveratrol and baseline was defined as DMSO readings in 64 wells. The data was normalized to positive and negative controls, and IC_{50} values were obtained using the average normalized luminescence intensity of 2 wells per concentration and a nonlinear variable slope four-parameter regression curve fitting model in Prism 6 (GraphPad Software Inc.).

To determine IC_{50} for luciferase inhibition in the third round of screening (631 compounds resourced from powder), the background for the luciferase inhibition was defined as average luminescence of the 16 wells with luciferase Inhibitor-I at single concentration of 10µM, and the baseline was defined as average luminescence of the 152 well with 0.5% DMSO. The data was normalized against positive and negative controls and IC_{50} were determined with Levenberg-Marquardt algorithm for curve fitting of the dose response data (fit parameters as Min = 0.0, Max ≥ 80.0, Slope ≥ 0.0) using CDD Vault from Collaborative Drug Discovery (Burlingame, CA. www.collaborativedrug.com). All data in this study were archived using the CDD Vault from Collaborative Drug Discovery (Burlingame, CA. www.collaborativedrug.com).

P. falciparum Dd2 asexual blood stage (*ABS-Sybr*) assay

P. falciparum Dd2 strain was cultured as described above in Culturing Asexual Erythrocytic-Stage *P. falciparum* parasites. For high throughput screening, 40nL of compounds in 1:3 serial dilutions in DMSO (0.5% final DMSO concentration per well) was transferred with Acoustic Transfer System (ATS) (Biosero) into black, clear-bottom 1536-well plates (custom GNF-mold ref# 789092-F, Greiner Bio-One). Artemisinin (5µM) at single concentration was used as positive and 0.5% DMSO was used as negative control. A parasite suspension with 0.3% parasitemia and 2.5% hematocrit in screening medium (SM) (RPMI 1640 with l-glutamine, without phenol red (Life Technologies, CA) supplemented with 0.2% AlbuMAX II lipid-rich BSA, 0.014mg/mL hypoxanthine, 3.4mM NaOH, 38.4mM Hepes, 0.2% glucose, 0.2% sodium bicarbonate, and 0.05mg/mL gentamicin) was prepared and dispensed into the 1536-well black, clear bottom plates with pre-spotted compounds using the MultiFloTM Microplate dispenser (BioTek) at volume 8µl per well. The plates containing the compounds and parasites were

incubated at 37°C for 72 h with water-soaked tissue in a double closed Ziploc bag gassed with 1% oxygen, 3% carbon dioxide, 96% nitrogen.

Fluorescence quantification *P. falciparum* asexual blood stage parasites (*ABS-Sybr*)

After incubation at 37°C for 72 h, the *P. falciparum* erythrocytic forms growth was assessed by fluorescence measurement as follows: detection reagent mixture (10x SYBR Green I (Invitrogen) in Lysis buffer (20mM Tris/HCl, 5mM EDTA, 0.16% Saponin wt/vol, 1.6% Triton X vol/vol) was added using MultiFloTM Microplate dispenser (BioTek) at volume 2µl per well. The plates were incubated in the dark at RT for 24 h. The fluorescence was read from the bottom of the plates by using the EnVision® Multilabel Reader (PerkinElmer) (485 nm excitation, 530 nm emission).

IC_{50} determination for *P. falciparum ABS-Sybr*

The background for the EF inhibition was defined as average fluorescence of the 16 wells with Artemisinin at single concentration of 5µM, and the baseline was defined as average fluorescence of the 96 well with 0.5% DMSO. The data was normalized as a percentage of negative control (0.5% DMSO) and IC_{50} were determined with Levenberg-Marquardt algorithm for curve fitting of the dose response data (fit parameters as Min = 0.0, Max ≥ 80.0, Slope ≥ 0.0) using CDD Vault from Collaborative Drug Discovery (Burlingame, CA. www.collaborativedrug.com) All data in this study were archived using the CDD Vault from Collaborative Drug Discovery (Burlingame, CA. www.collaborativedrug.com).

P. vivax high content liver stage assay

The *P. vivax* liver stage assay used in this study has been described elsewhere(*38*). In brief, collagen coated 384-well plates (Grenier, Monroe, NC, USA) were seeded with 18,000 live primary human hepatocytes (BioIVT, Westbury, NY, USA) 1-4 days prior to sporozoite infection. Plating media containing serum (BioIVT) and supplemented with 50µg/mL penicillin-streptomycin, 100µg/mL of neomycin, and 10µg/mL of gentamicin was changed prior to sporozoite infection, the day after sporozoite infection, and between daily drug additions. The Human Subjects protocols for this study was approved by the Institutional Ethics Committee of the Thai Ministry of Public Health and the Oxford Tropical Medicine Ethical Committee, Oxford University, England (TMEC 14-016 and OxTREC 40-14). Blood containing at least 5 *P. vivax* gametocytes per 500 leukocytes (counted by Giemsa-stained blood smear) was collected by venipuncture into heparin tubes from malaria patients reporting to malaria clinicals after giving written informed consent. Collected blood from anonymized donors was pelleted and serum replaced (1 volume to pellet volume) with O+ serum and then fed to day 5-7 old female mosquitoes via artificial membrane feeder (Hemotek, Great Harwood, England)(*39*). Fed mosquitoes were separated from unfed and kept on 10% sucrose via wetted cotton in a environmental chamber set to maintain 26 ºC and 80% humidity. Salivary glands were dissected into Schneiders' insect medium (pH at 7.1) at day 14 post feed, counted by hemocytometer, diluted in media, and 7,000 sporozoites added to each assay well prior to centrifugation of the assay plate at 200 G for 5min(*40*). Compounds were supplied pre-plated at 10mM in DMSO (Evotec). To add compound a custom-ordered hand-held pin-tool, designed to transfer 40nL (VP Scientific, San Diego, CA, USA), was dipped in drug plates and then inserted into assay plates with 40µL media, achieving a 1000x dilution of drug. The pin-tool was washed and blotted

twice into each of 5 washing solutions: 50% DMSO, 70% ethanol, VP clean solution, water, and methanol, prior to transfer for additional drug plates.

Each assay plate contained 12 DMSO control wells, 8 KDU691 positive controls wells, and 8 atovaquone reference wells. At 6 days post infection cultures were fixed with 4% paraformaldehyde for 10 min and washed twice with PBS prior to overnight staining at 4 ºC with recombinantly expressed mouse anti-PvUIS4 diluted to 40 ng/mL in dilution buffer (0.03% TritonX-100 and1 % (w/v) BSA in 1× PBS). Following three PBS washes, the primary stain was detected with goat anti-mouse Alexa Fluor® 488 (Molecular Probes, ThermoFisher, Waltham, MA, USA) diluted to 2µg/mL in dilution buffer, and counterstained with 10µg/mL Hoechst for 1hr at 37ºC. Assay plates were imaged and analyzed by the Operetta Imaging System and Harmony software 4.1 (Perkin Elmer, Waltham, MA, U.S.A.). Images were acquired with FITC, DAPI, and brightfield channels using a 20× objective. Host cell nuclei were quantified using DAPI counts while parasites were identified and classified by growth area. Raw parasite growth metrics were loaded into CDD Vault for normalization and hit identification. *Z*' factor for controls were calculated for both biological replicates of all three plates containing 631 screen compounds(*41*). Schizont count Z' ranged from -0.02 to 0.35 and schizont growth area -0.39 to 0.19.

Computational compound clustering

All 538,273 compounds in the primary screen were hierarchically clustered based on structure similarity using JChem fingerprint. Compound clusters are separated based on a minimum Tanimoto coefficient requirement of 0.85. Primary hits are defined as compounds with >82.98% inhibition. Hit enrichment probability of each cluster is estimated by the redundant siRNA activity (RSA)(*42*) p-value using inhibition percentage.

Metabolomic profiling

Metabolic response to selected compounds was determined using whole cell hydrophilic extraction followed by ultra-high precision liquid chromatography mass-spectrometry (UHPLC-MS) as previously described(*19*). Briefly, synchronous, staged trophozoite infected cells (24-36 hpi) were magnetically separated from culture. This was followed by quantification of cells via hemocytometry, subsequent aliquoting of $1x10^8$ cells a well, and recovery for 1-2 hours under incubation. The wells were then treated with $10xIC_{50}$ of compound for 2.5 hours, with each condition performed in triplicate, and included a no drug control and a positive control of atovaquone in each experiment. Cells were then collected by centrifugation and washed using PBS. Hydrophilic extraction of metabolites was performed using 90% methanol containing isotopically-labeled aspartate (0.5µM) as an internal standard, and dried under nitrogen(*20*). Metabolite extracts were then measured by UHPLC-MS and the subsequent data was analyzed as previously described(*20*). The averaged metabolite data for each drug treatment was directly compared to an untreated control and the resulting fold-changes (Data S5) were hierarchically clustered based on a Pearson distance and visualized using MetaPrint self-organizing maps (*20, 44*).

Low throughput ScDHODH assay and heatmap generation

Drug susceptibility in the Dd2-attB and Dd2-ScDHODH cell lines was measured by growth assay as previously reported(*44*). Briefly, synchronized ring stage parasites were cultured in the presence of twelve-point serial dilutions of the test compounds in triplicate in 384-well black clear-bottom plates for 72 h. Lysis buffer and SYBR Green I fluorescent dye was added as above, and after overnight incubation at room temperature fluorescence was read on a Molecular Devices SpectraMax M5 plate reader (bottom read, excitation and emission wavelengths of 485 nm and 530 nm). IC_{50} values were calculated using a nonlinear regression curve fit in Prism Software version 7 (GraphPad). The assay was replicated four to eight times.

The extent of cross-resistance was determined by dividing the IC_{50} of the Dd2-ScDHODH line by the IC_{50} of the parent Dd2-attB strain, and taking the log_{10} of this IC_{50} ratio. These values were visualized on a heatmap generated in Prism Software version 7 (GraphPad). In cases where the IC_{50} value was outside the range of the highest dose tested, the maximum dose tested (10 µM) was used as a placeholder value to generate the heatmap.

1536 well ScDHODH assay with proguanil (high-throughput mode)

To identify and differentiate between Cytb and DHODH inhibitors in the set of 631 compounds from the third round of screening, drug susceptibility in D10-attb and D10-ScDHODH blood-stage parasites in the presence and absence of proguanil was determined. The assay was performed in 1536-well format with the same experimental conditions as described in the section: *P. falciparum* Dd2 Asexual blood stage assay (*ABS-Sybr*), with the exception that the compounds were tested in eight-point serial dilutions and in duplicate. In the absence of proguanil (1µM), the D10-ScDHODH strain is resistant to mitochondrial electron transport chain inhibitors. In the presence of 1µM proguanil, sensitivity to Cytb inhibitors is restored but resistance to DHODH inhibitors remains. This distinction allows identification of the specific mitochondrial electron transport chain target for each compound.

In vitro evolution experiments

For MMV1432711, three independent resistant cultures were created by exposing a clonal Dd2 strain to sublethal drug pressure for 16 weeks at a starting concentration of 30nM using the "ramp-up" method to a final concentration of 600nM. Parasite growth rates were monitored daily and IC_{50} curves collected every 2 weeks. Dose-response assay showed a shift of ~8-fold for the two replicates compared to the parental clone (106nM and 149.6nM relative to the parental value of 15.48nM). These replicates also showed a phenotypic change since MMV1432711 typically arrested parasites in late trophozoite phase. DNA was isolated from the resistant nonclonal cultures and submitted for whole genome sequencing.

For MMV1454442, approximately 5×10^8 Dd2 parasites in three independent flasks were treated at an initial concentration of 2µM MMV1454442 until parasites could not be seen by microscopy. Following treatment, compound pressure was removed and cultures were allowed to recover in the absence of drug. Once healthy parasites were seen and parasitemia reached ~2%, compound pressure was reinstated at an increased dose of 3µM. Cultures were again allowed to recover in the absence of compound and then re-pressured with 5µM MMV1454442. Parasites that recovered from the 5µM treatment were phenotypically resistant by dose-response assay. Resistant parasites were cloned by limiting dilution in the absence of drug pressure. Individual clones were then phenotyped again and submitted for whole genome sequencing.

For MMV1427995, approximately 10^9 parasites were exposed to 1.3μM of the compound (approximately 4x IC_{50}). Parasites that recrudesced on day 21 showed no IC_{50} shift and were then subjected to a ramping procedure starting at 650nM and gradually building up to 1.5μM (approximately 4.75 x IC_{50}) over a course of 40 days. The resulting resistant populations were cloned as described above and, phenotyped for MMV142795 susceptibility and clones with varying IC_{50} shifts were submitted for whole genome sequencing.

DNA Sequence analysis of MMV1454442, MMV1432711, and MMV1427995-resistant clones

Infected RBCs were washed with 0.05% saponin and genomic DNA (gDNA) was isolated using the DNeasy Blood and Tissue Kit (Qiagen) standard protocol. gDNA was tagmented and amplified with the Nextera XT Kit (Cat. No FC-131-1024, Illumina) following the standard dual index protocol to prepare the sequencing libraries. Samples were sequenced on the Illumina HiSeq 2500 in RapidRun mode, generating paired-end reads 100bp in length. Following sequencing, reads were aligned to the *P. falciparum* reference genome (PlasmoDB v13.0) using the previously described Platypus pipeline(*45*). Sequences contained an average of 24,811,649 reads of 100bp with 95.6% mapping to the reference genome. An average coverage of 72.88x coverage was obtained with 95% of the genome covered by 5 or more reads. SNVs and INDELs were called using GATK's HaplotypeCaller and filtered using GATK's best practice recommendations(*46*). CNVs were detected using GATK's DiagnoseTargets, which computes average coverage using an input of gene lengths and locations as the analyzed intervals. Genes with known high variability in copy numbers, such as those in the *var*, *rifin*, and *stevor* families, were removed from the interval list to avoid potential misalignment. Read coverage was normalized first by log transforming and centering genes to the mean and then centering sequence samples to the sample means. CNVs were called if they contained >3x coverage compared to the average over the length of the CNV and were visually confirmed in Integrative Genomics Viewer (IGV).

Figure S1

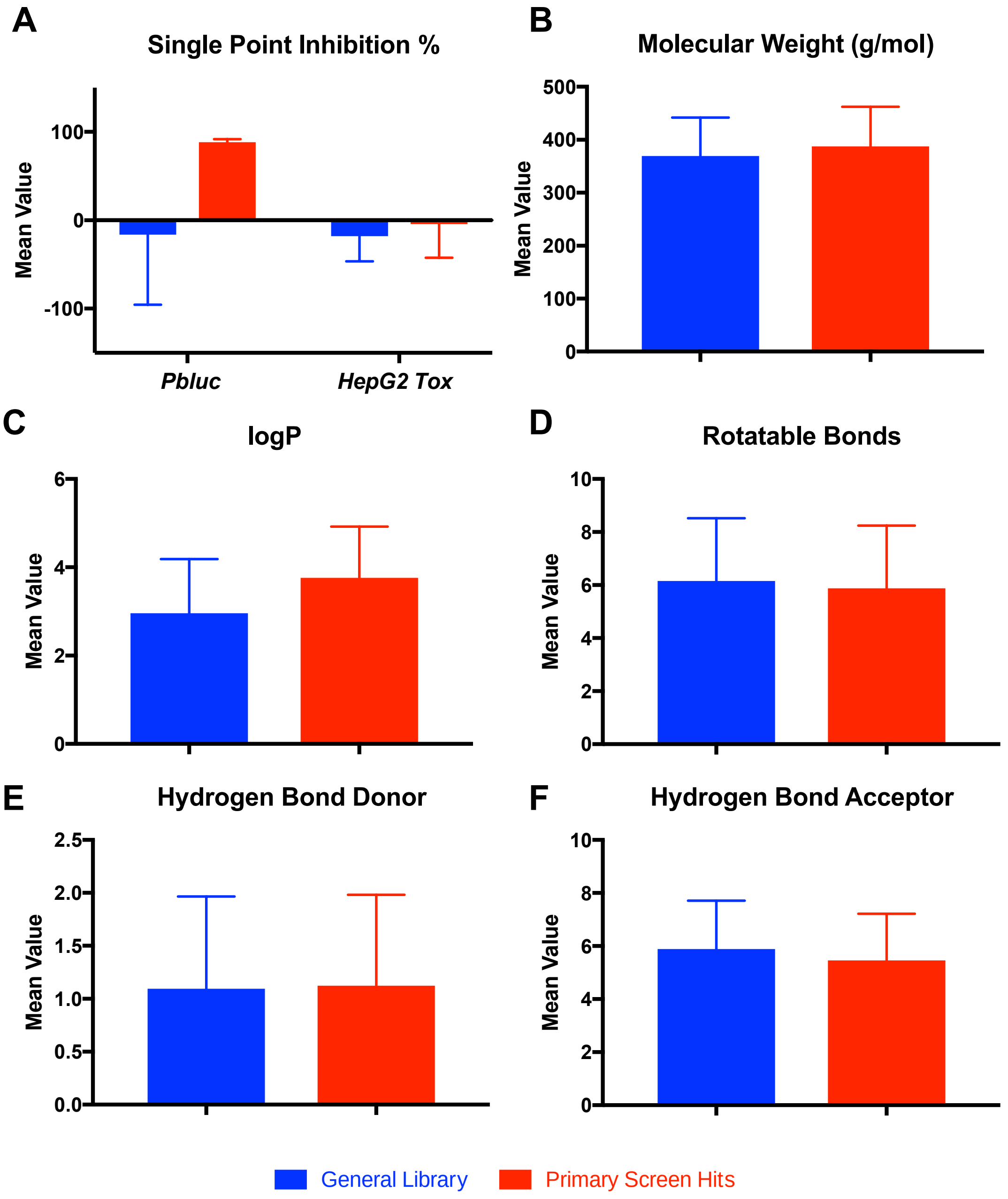

Figure S1. Library characteristics. Differences between the initial library (538,273 compounds, blue) and hits (9,989, red). Comparison of library characteristics between full compound library (blue) and primary screen hits (red). Each bar represents the mean value for each characteristic with error bars indicating the standard deviation, as reported in Tables S1 and S2.

Figure S2

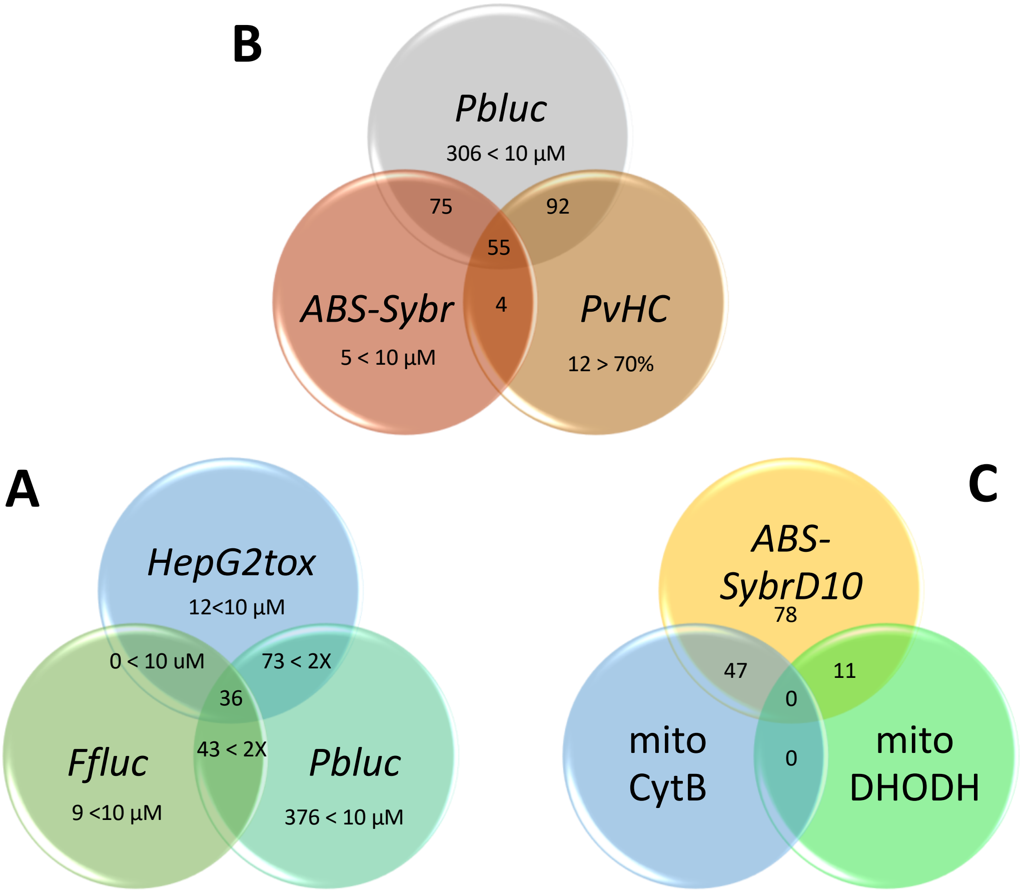

Figure S2. Venn Diagram showing relationships between activities in the Repurchased Validation Set (631 compounds),

Figure S3

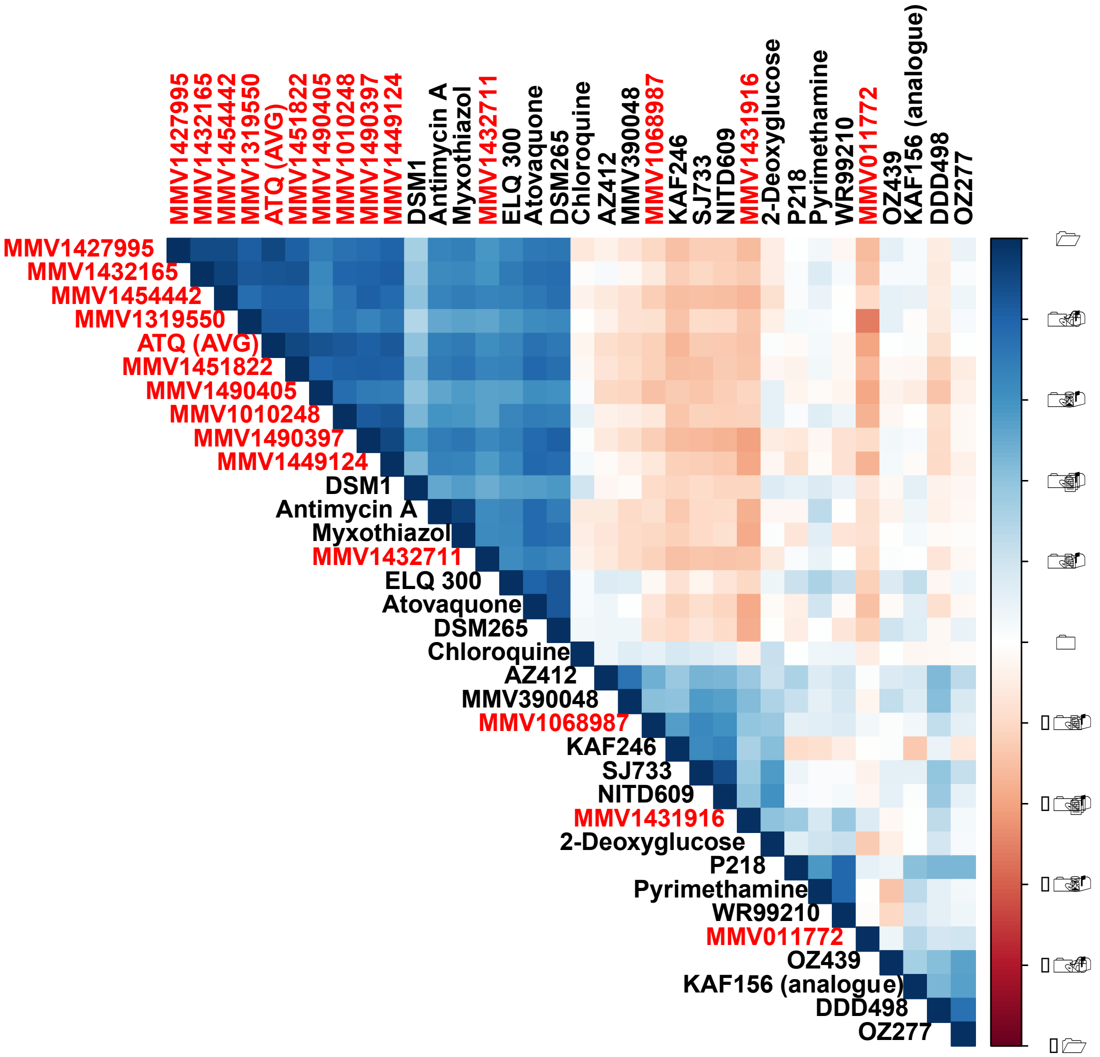

Figure

Figure S3. Heatmap of metabolomic data. Displayed is a heatmap representing compounds' correlation coefficients of $\log_2$ fold changes for all metabolites. Compounds denoted in red are primary to this study and are compared to those previously published (*20*). Compounds are clustered by Ward hierarchical sorting.

Figure S4

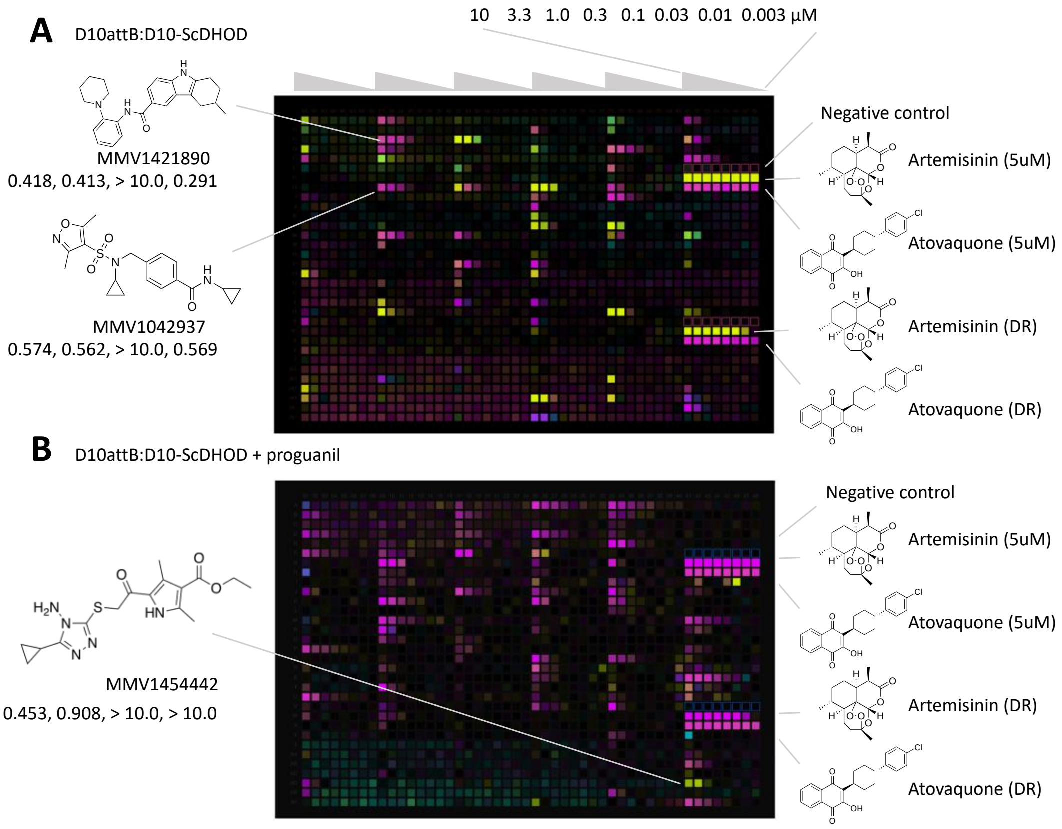

Figure S4. 1536-well ScDHOD plate images (72 hour, SYBR green). The image shows the primary 8-pt dose response data with a pseudocolor comparison of D10attB and D10-ScDHOD (A) and the same in the presence of proguanil (B). In A, purple horizontal strips represent mitochondrial inhibitors, and yellow, non-mitochondrial inhibitors dispensed at final concentrations shown at the top. Horizontal groups of eight with no signal are compounds that are only active in the *Pbluc* assay. The eight negative control wells are wells that were not treated with inhibitor. Artemisinin and atovaquone were added to each plate as a positive control. In B, yellow indicates compounds with a high likelihood of being DHODH inhibitors. Two different plates from one of two replicates are shown.

Figure S5

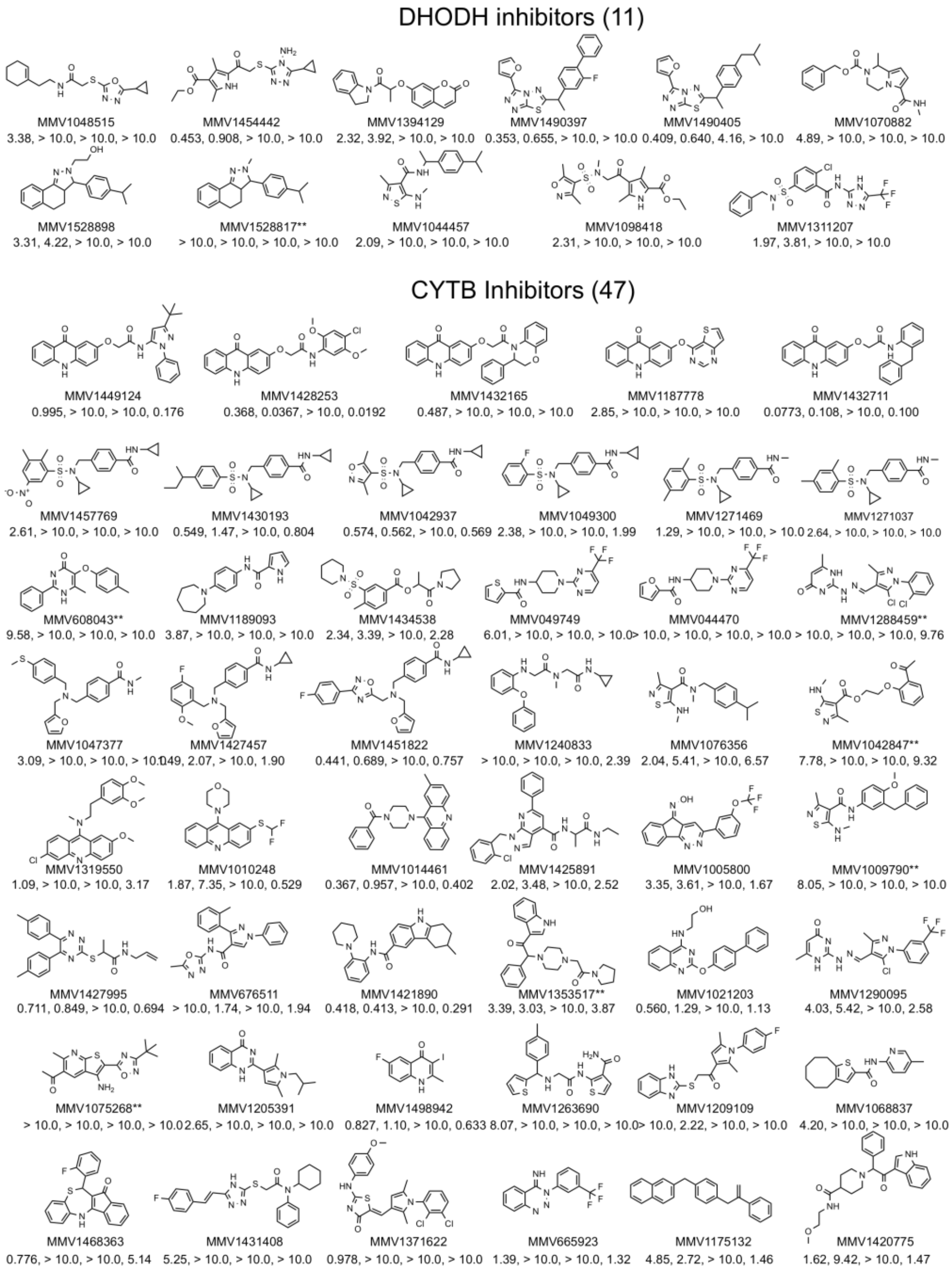

Figure S5. Structures of likely mitochondrial inhibitors. IC_{50} values (µM) are given below the compound names and are for D10-attB, D10-ScDHODH, D10-attB + 1µM proguanil, and D10-ScDHODH + proguanil. In cases where the IC_{50} values shows > 10µM (no fit), curves (See Data S7) were visually inspected. Compounds marked with ** showed only partial inhibition at the highest concentration but were judged to be likely mitochondrial inhibitors based on a strong loss of signal in the ScDHODH ABS-Sybr 72-hour dose response experiment relative to the D10 control line. The results are from biological replicates performed on separate days.

Table S1.

Property	Count	Mean	Max	Min	Std. Dev.
Pbluc (single point inhibition, %age)	538,273	-16.3	100	-1290	79.3
HepG2tox (single point inhibition, %age)	179,600	-17.8	97	-362	28.6
logP	538,273	2.96	11.9	-4.72	1.22
Molecular weight	538,273	369	1298	60.06	72.6
Hydrogen bond donor	538,273	1.01	24	0	0.870
Hydrogen bond acceptor	538,273	5.89	40	0	1.83
Rotatable bonds	538,273	6.15	45	0	2.36

Table S1. General Library Characteristics

Table S2.

Property	Count	Mean	Max	Min	Std. Dev.
Pbluc (single point inhibition, %age)	9,944	88.3	100	82.98	3.66
HepG2tox (single point inhibition, %age)	3,006	-4.14	50	-247	38.2
logP	9,944	3.76	9.944	-1.44	1.16
Molecular weight	9,944	387.9	985.1	136.2	74.3
Hydrogen bond donor	9,944	1.123	8	0	0.859
Hydrogen bond acceptor	9,944	5.46	20	0	1.76
Rotatable bonds	9,944	5.881	24	0	2.36

Table S2. Primary Screen Hit Characteristics. *HepG2tox* data was only available for 3,006 compounds.

Table S3.

Compound	Dd2-attB		Dd2-ScDHODH		Fold Change IC_{50} (Dd2-ScDHODH/Dd2-attB)
	IC_{50} ± std. dev. (μM)	n[a]	IC_{50} ± std. dev. (μM)	n[a]	
**MMV1068987**	0.287 ± 0.12	4	0.237 ± 0.059	4	0.83
**MMV1490397**	0.149 ± 0.044	8	> 10.0	8	> 67
**MMV1449124**	0.109 ± 0.053	4	> 10.0	4	> 92
**MMV1432711**	0.00839 ± 0.0023	4	> 10.0	4	> 1200
**MMV1010248**	0.201 ± 0.081	4	> 10.0	4	> 50
**MMV1432165**	0.0310 ± 0.0079	4	> 10.0	4	> 320
**MMV1451822**	0.0993 ± 0.017	4	> 10.0	4	> 100
**MMV1490405**	0.0681 ± 0.020	4	> 10.0	4	> 150
**MMV1454442**	0.0997 ± 0.018	4	> 10.0	4	> 100
**MMV1319550**	0.247 ± 0.066	4	> 5.0	4	> 20
**MMV1427995**	0.101 ± 0.025	4	> 10.0	4	> 99
**MMV011772**	0.504 ± 0.061	4	0.612 ± 0.200	4	1.2
**Chloroquine**	0.131 ± 0.012	4	0.134 ± 0.0075	4	1.0
**Mefloquine**	0.00771 ± 0.0029	8	0.00999 ± 0.0038	8	1.3
**Amodiaquine**	0.0101 ± 0.0019	8	0.0102 ± 0.0027	8	1.0
**Artemether**	0.00962 ± 0.0048	8	0.00981 ± 0.0037	8	1.0
**Atovaquone**	0.000285 ± 0.00010	8	> 0.050	8	> 180
**Piperaquine**	0.0154 ± 0.0058	8	0.0124 ± 0.004.8	8	0.81

Table S3. Mitochondrial inhibition without proguanil rescue. Dose response activity of compounds in wild-type Dd2-attB and transgenic Dd2-ScDHODH parasite lines. [a]number of bioreplicate assays

Table S4.

Compound	Dd2 IC_{50} ± std. dev. (μM)	Dd2+PG IC_{50} ± std. dev. (μM)	DHODH IC_{50} ± std. dev. (μM)	DHODH+PG IC_{50} ± std. dev. (μM)	Ratio DHODH/ Dd2	Ratio DHODH+ PG/Dd2
MMV1432165	0.433 ± 0.104	0.405 ± 0.168	21.1 ± 10.7	0.528 ± 0.167	42.7	1.3
MMV1451822	0.165 ± 0.0613	0.0870 ± 0.0506	>50	0.166 ± 0.0151	>215	1.1
MMV1490405	0.509 ± 0.0400	0.592 ± 0.228	16.3 ± 6.22	11.9 ± 4.34	31.5	23.4
MMV1490397	0.447 ± 0.156	0.453 ± 0.0529	33.8 ± 16.5	23.0 ± 7.11	90.9	53.1
MMV1454442	0.970 ± 0.373	0.816 ± 0.338	>50	>50	>36	>36
MMV1427995	0.943 ± 0.180	0.400 ± 0.314	12.7 ± 1.27	0.536 ± 0.129	13.7	0.6
MMV011772	0.706 ± 0.428	0.848 ± 0.0871	0.885 ± 0.273	1.10 ± 0.589	1.0	1.2

Table S4. Mitochondrial inhibition data with proguanil rescue. Shown is dose response activity of select compounds in Dd2-scDHODH parasites with addition of proguanil (PG), which exhibits a synergistic effect specifically with Cytbc1 inhibitors. Compounds with a DHODH+PG/Dd2 ratio of 1 are Cytbc1 inhibitors (indicating a restoration of sensitive phenotype) while those with a ratio >3 (resistance phenotype) are DHODH inhibitors.

Table S5.

Clone Name	Average Clone IC_{50} ± std. dev. (μM)	Parent Clone IC_{50} ± std. dev. (μM)	Fold Change IC_{50}
MMV1454442-1F1	0.611	0.130	4.7
MMV1454442-1F7	0.427	0.130	3.3
MMV1454442-1F9	0.418	0.130	3.2
MMV1454442-2C3	0.588	0.130	4.5
MMV1454442-2C9	0.663	0.130	5.1
MMV1454442-2E11	0.251	0.130	1.9
MMV1454442-3E5	0.345	0.130	2.7
MMV1454442-3E10	0.377	0.130	2.9
MMV1454442-3F10	1.216	0.130	9.4
MMV1432711-bulk1	0.106	0.0155	6.9
MMV1432711-bulk2	0.150	0.0155	9.7
MMV1432711-bulk3	0.0129	0.0155	0.8
MMV1427995-bulk1	0.588 ± 0.081	0.210 ± 0.046	2.8
MMV1427995-bulk2	0.413 ± 0.060	0.210 ± 0.046	2.0
MMV142799-2C11	0.547 ± 0.105	0.210 ± 0.046	2.6
MMV1427995-2F4	0.493 ± 0.088	0.210 ± 0.046	2.3

Table S5. Clone characteristics for resistant *P. falciparum* lines. IC_{50} of each compound was determined in dose-response format using a SYBR Green-I cell proliferation assay (*ABS-Sybr*).

Table S6.

Clone Name	Total Base Pairs Sequenced	Mean Coverage (X)	Percent Bases Aligned to Reference	Percent Bases with 5 or More Reads
MMV1454442-1F1	1,793,576,237	76.9	97.7	96.2
MMV1454442-1F7	1,644,003,801	70.5	97.5	95.3
MMV1454442-1F9	1,890,300,497	81.0	97.4	95.9
MMV1454442-2C3	1,181,219,186	50.6	96.4	93.9
MMV1454442-2C9	1,708,681,968	73.2	94.6	95.5
MMV1454442-2E11	2,071,599,344	88.8	95.5	96.2
MMV1454442-3E5	3,266,145,971	140.0	95.7	97.3
MMV1454442-3E10	1,124,845,257	48.2	97.3	92.7
MMV1454442-3F10	1,302,880,841	55.8	96.1	95.0
MMV1454442-parent	1,022,068,015	43.8	97.9	91.9
MMV1432711-bulk1	1,432,276,721	61.4	97.7	95.9
MMV1432711-bulk2	1,297,956,846	55.6	97.2	93.5
MMV1432711-bulk3	1,386,328,144	59.4	97.4	95.7
MMV1432711-parent	1,237,459,126	53.0	97.2	94.9
MMV1427995-bulk1	2,779,543,707	119.1	98.3	97.1
MMV1427995-bulk2	2,668,328,673	114.4	98.4	97.2
MMV1427995-2C11	2,345,202,988	100.5	98.1	96.5
MMV1427995-2F4	2,489,912,105	106.7	98.4	97.4
MMV1427995-parent	1,861,423,637	79.8	92.9	96.0

Table S6. Whole genome sequencing statistics for resistant *P. falciparum* lines and their corresponding sensitive parents. Sequences have been deposited in the shortread sequence archive under accession number SRP134381.

Table S7.

Clone Name	CNV Start	CNV End	Number of Genes Amplified in CNV	Target or Resistance Gene in Amplified Region
MMV1454442-2C3	PF3D7_0602700	PF3D7_0603300	7	*pfdhodh* (PF3D7_0603300)
MMV1454442-2C9	PF3D7_0602700	PF3D7_0603300	7	*pfdhodh* (PF3D7_0603300)
MMV1454442-2E11	PF3D7_0602700	PF3D7_0603300	7	*pfdhodh* (PF3D7_0603300)
MMV1454442-3E5	PF3D7_0602700	PF3D7_0603300	7	*pfdhodh* (PF3D7_0603300)
MMV1454442-3E10	PF3D7_0602700	PF3D7_0603300	7	*pfdhodh* (PF3D7_0603300)
MMV1454442-3F10	PF3D7_0602700	PF3D7_0603300	7	*pfdhodh* (PF3D7_0603300)
MMV1427995-bulk1	PF3D7_0521900	PF3D7_0523200	14	*pfmdr1* (PF3D7_0523000)

Table S7. Identified copy number variants in evolved, compound-resistant clones. Variants were detected as described in methods and are based on increased read coverage per gene in evolved clone relative to sensitive parent. Start and end positions on the indicated chromosome are approximate and include the indicated gene.

Table S8.

**Compound**	**Purity (%)**
MMV1432165	92.10%
MMV1451822	96.00%
MMV1068987	96.40%
MMV1490405	100.00%
MMV1490397	99.20%
MMV1449124	90.90%
MMV1432711	97.80%
MMV1454442	88.20%
MMV1010248	17.20%
MMV1319550	73.60%
MMV1427995	94.30%
MMV1431916	76.00%
MMV011772	100.00%

Table S8. Compound purity assessed by mass spectrometry.

Data S1. (separate file)

Compounds previously identified in high content imaging assays *(9)*. The 197 compounds shared with this study were initially identified in an *P. falciparum* ABS assay (*ABS-Sybr*) and then retested against *Plasmodium. yoelii* hepatic stages *(9)*. The file shows data from the previous publication, including whether the compound was a “hit” in the *P. yoelii* high content liver stage assay, whether it was selected for powder-resupply dose response testing in the high content *P. yoelii* assay (based on percent inhibition and compound availability), as well as the primary *Pbluc* and *HepG2tox* percent inhibition data from this study (attached Excel spreadsheet, 197 compounds).

Data S2. (separate file)

Clustering results. The file contains names, structures and cluster number for all compounds that are structurally similar to compounds with >83% inhibition in *Pbluc* (attached Excel spreadsheet, 17,384 compounds). Also included are structure displays for 405 compounds from 68 clusters that show a p-value <= 0.05, cluster size >= 4 and hit fraction >= 0.75 are which are presented in Fig. 2.

Data S3. (separate file)

Reconfirmation Set. File contains names, structures, and % inhibition in primary screen as well as dose response in secondary tests (attached Excel spreadsheet 9,989 compounds)

Data S4. (separate file)

Repurchased Validation Set (631 Compounds). Compounds selected for third round characterization (attached Excel spreadsheet, 631 compounds)

Data S5. (separate file)

Metabolomic data. The file contains raw metabolomic data used to create metaprints (attached Excel spreadsheet).

Data S6. (separate file)

Sequencing Data. The file contains whole genome sequencing variant detection files (attached Excel spreadsheet)

Data S7. (separate file)

Mitochondrial inhibition data. Dose response curves for compounds active in the D10-ScDHODH high-throughput (1536-well) assay. The attached Excel spreadsheet shows values for the 631 compounds (Repurchased Validation Set) plus two controls (atovaquone and artemisinin)

References and Notes

1. World Health Organization (WHO), *World Malaria Report 2017* (WHO, 2017).

2. B. Blasco, D. Leroy, D. A. Fidock, Antimalarial drug resistance: Linking *Plasmodium falciparum* parasite biology to the clinic. *Nat. Med.* **23**, 917–928 (2017). doi:10.1038/nm.4381 Medline

3. M. A. Phillips, J. N. Burrows, C. Manyando, R. H. van Huijsduijnen, W. C. Van Voorhis, T. N. C. Wells, Malaria. *Nat. Rev. Dis. Primers* **3**, 17050 (2017). doi:10.1038/nrdp.2017.50 Medline

4. E. L. Flannery, D. A. Fidock, E. A. Winzeler, Using genetic methods to define the targets of compounds with antimalarial activity. *J. Med. Chem.* **56**, 7761–7771 (2013). doi:10.1021/jm400325j Medline

5. J. R. Matangila, P. Mitashi, R. A. Inocêncio da Luz, P. T. Lutumba, J. P. Van Geertruyden, Efficacy and safety of intermittent preventive treatment for malaria in schoolchildren: A systematic review. *Malar. J.* **14**, 450 (2015). doi:10.1186/s12936-015-0988-5 Medline

6. D. Plouffe, A. Brinker, C. McNamara, K. Henson, N. Kato, K. Kuhen, A. Nagle, F. Adrián, J. T. Matzen, P. Anderson, T. G. Nam, N. S. Gray, A. Chatterjee, J. Janes, S. F. Yan, R. Trager, J. S. Caldwell, P. G. Schultz, Y. Zhou, E. A. Winzeler, In silico activity profiling reveals the mechanism of action of antimalarials discovered in a high-throughput screen. *Proc. Natl. Acad. Sci. U.S.A.* **105**, 9059–9064 (2008). doi:10.1073/pnas.0802982105 Medline

7. W. A. Guiguemde, A. A. Shelat, D. Bouck, S. Duffy, G. J. Crowther, P. H. Davis, D. C. Smithson, M. Connelly, J. Clark, F. Zhu, M. B. Jiménez-Díaz, M. S. Martinez, E. B. Wilson, A. K. Tripathi, J. Gut, E. R. Sharlow, I. Bathurst, F. El Mazouni, J. W. Fowble, I. Forquer, P. L. McGinley, S. Castro, I. Angulo-Barturen, S. Ferrer, P. J. Rosenthal, J. L. Derisi, D. J. Sullivan, J. S. Lazo, D. S. Roos, M. K. Riscoe, M. A. Phillips, P. K. Rathod, W. C. Van Voorhis, V. M. Avery, R. K. Guy, Chemical genetics of *Plasmodium falciparum*. *Nature* **465**, 311–315 (2010). doi:10.1038/nature09099 Medline

8. F. J. Gamo, L. M. Sanz, J. Vidal, C. de Cozar, E. Alvarez, J.-L. Lavandera, D. E. Vanderwall, D. V. S. Green, V. Kumar, S. Hasan, J. R. Brown, C. E. Peishoff, L. R. Cardon, J. F. Garcia-Bustos, Thousands of chemical starting points for antimalarial lead identification. *Nature* **465**, 305–310 (2010). doi:10.1038/nature09107 Medline

9. S. Meister, D. M. Plouffe, K. L. Kuhen, G. M. C. Bonamy, T. Wu, S. W. Barnes, S. E. Bopp, R. Borboa, A. T. Bright, J. Che, S. Cohen, N. V. Dharia, K. Gagaring, M. Gettayacamin, P. Gordon, T. Groessl, N. Kato, M. C. S. Lee, C. W. McNamara, D. A. Fidock, A. Nagle, T. G. Nam, W. Richmond, J. Roland, M. Rottmann, B. Zhou, P. Froissard, R. J. Glynne, D. Mazier, J. Sattabongkot, P. G. Schultz, T. Tuntland, J. R. Walker, Y. Zhou, A. Chatterjee, T. T. Diagana, E. A. Winzeler, Imaging of *Plasmodium* liver stages to drive next-generation antimalarial drug discovery. *Science* **334**, 1372–1377 (2011). doi:10.1126/science.1211936 Medline

10. J. Swann, V. Corey, C. A. Scherer, N. Kato, E. Comer, M. Maetani, Y. Antonova-Koch, C. Reimer, K. Gagaring, M. Ibanez, D. Plouffe, A.-M. Zeeman, C. H. M. Kocken, C. W. McNamara, S. L. Schreiber, B. Campo, E. A. Winzeler, S. Meister, High-throughput

luciferase-based assay for the discovery of therapeutics that prevent malaria. *ACS Infect. Dis.* **2**, 281–293 (2016). doi:10.1021/acsinfecdis.5b00143 Medline

11. K. L. Kuhen, A. K. Chatterjee, M. Rottmann, K. Gagaring, R. Borboa, J. Buenviaje, Z. Chen, C. Francek, T. Wu, A. Nagle, S. W. Barnes, D. Plouffe, M. C. S. Lee, D. A. Fidock, W. Graumans, M. van de Vegte-Bolmer, G. J. van Gemert, G. Wirjanata, B. Sebayang, J. Marfurt, B. Russell, R. Suwanarusk, R. N. Price, F. Nosten, A. Tungtaeng, M. Gettayacamin, J. Sattabongkot, J. Taylor, J. R. Walker, D. Tully, K. P. Patra, E. L. Flannery, J. M. Vinetz, L. Renia, R. W. Sauerwein, E. A. Winzeler, R. J. Glynne, T. T. Diagana, KAF156 is an antimalarial clinical candidate with potential for use in prophylaxis, treatment, and prevention of disease transmission. *Antimicrob. Agents Chemother.* **58**, 5060–5067 (2014). doi:10.1128/AAC.02727-13 Medline

12. C. W. McNamara, M. C. S. Lee, C. S. Lim, S. H. Lim, J. Roland, O. Simon, B. K. Yeung, A. K. Chatterjee, S. L. McCormack, M. J. Manary, A. M. Zeeman, K. J. Dechering, T. S. Kumar, P. P. Henrich, K. Gagaring, M. Ibanez, N. Kato, K. L. Kuhen, C. Fischli, A. Nagle, M. Rottmann, D. M. Plouffe, B. Bursulaya, S. Meister, L. Rameh, J. Trappe, D. Haasen, M. Timmerman, R. W. Sauerwein, R. Suwanarusk, B. Russell, L. Renia, F. Nosten, D. C. Tully, C. H. M. Kocken, R. J. Glynne, C. Bodenreider, D. A. Fidock, T. T. Diagana, E. A. Winzeler, Targeting *Plasmodium* PI(4)K to eliminate malaria. *Nature* **504**, 248–253 (2013). doi:10.1038/nature12782 Medline

13. B. Baragaña, I. Hallyburton, M. C. Lee, N. R. Norcross, R. Grimaldi, T. D. Otto, W. R. Proto, A. M. Blagborough, S. Meister, G. Wirjanata, A. Ruecker, L. M. Upton, T. S. Abraham, M. J. Almeida, A. Pradhan, A. Porzelle, T. Luksch, M. S. Martínez, T. Luksch, J. M. Bolscher, A. Woodland, S. Norval, F. Zuccotto, J. Thomas, F. Simeons, L. Stojanovski, M. Osuna-Cabello, P. M. Brock, T. S. Churcher, K. A. Sala, S. E. Zakutansky, M. B. Jiménez-Díaz, L. M. Sanz, J. Riley, R. Basak, M. Campbell, V. M. Avery, R. W. Sauerwein, K. J. Dechering, R. Noviyanti, B. Campo, J. A. Frearson, I. Angulo-Barturen, S. Ferrer-Bazaga, F. J. Gamo, P. G. Wyatt, D. Leroy, P. Siegl, M. J. Delves, D. E. Kyle, S. Wittlin, J. Marfurt, R. N. Price, R. E. Sinden, E. A. Winzeler, S. A. Charman, L. Bebrevska, D. W. Gray, S. Campbell, A. H. Fairlamb, P. A. Willis, J. C. Rayner, D. A. Fidock, K. D. Read, I. H. Gilbert, A novel multiple-stage antimalarial agent that inhibits protein synthesis. *Nature* **522**, 315–320 (2015). doi:10.1038/nature14451 Medline

14. N. Kato, E. Comer, T. Sakata-Kato, A. Sharma, M. Sharma, M. Maetani, J. Bastien, N. M. Brancucci, J. A. Bittker, V. Corey, D. Clarke, E. R. Derbyshire, G. L. Dornan, S. Duffy, S. Eckley, M. A. Itoe, K. M. J. Koolen, T. A. Lewis, P. S. Lui, A. K. Lukens, E. Lund, S. March, E. Meibalan, B. C. Meier, J. A. McPhail, B. Mitasev, E. L. Moss, M. Sayes, Y. Van Gessel, M. J. Wawer, T. Yoshinaga, A.-M. Zeeman, V. M. Avery, S. N. Bhatia, J. E. Burke, F. Catteruccia, J. C. Clardy, P. A. Clemons, K. J. Dechering, J. R. Duvall, M. A. Foley, F. Gusovsky, C. H. M. Kocken, M. Marti, M. L. Morningstar, B. Munoz, D. E. Neafsey, A. Sharma, E. A. Winzeler, D. F. Wirth, C. A. Scherer, S. L. Schreiber, Diversity-oriented synthesis yields novel multistage antimalarial inhibitors. *Nature* **538**, 344–349 (2016). doi:10.1038/nature19804 Medline

15. B. Franke-Fayard, C. J. Janse, M. Cunha-Rodrigues, J. Ramesar, P. Büscher, I. Que, C. Löwik, P. J. Voshol, M. A. M. den Boer, S. G. van Duinen, M. Febbraio, M. M. Mota, A.

P. Waters, Murine malaria parasite sequestration: CD36 is the major receptor, but cerebral pathology is unlinked to sequestration. *Proc. Natl. Acad. Sci. U.S.A.* **102**, 11468–11473 (2005). doi:10.1073/pnas.0503386102 Medline

16. M. A. Phillips, J. Lotharius, K. Marsh, J. White, A. Dayan, K. L. White, J. W. Njoroge, F. El Mazouni, Y. Lao, S. Kokkonda, D. R. Tomchick, X. Deng, T. Laird, S. N. Bhatia, S. March, C. L. Ng, D. A. Fidock, S. Wittlin, M. Lafuente-Monasterio, F. J. G. Benito, L. M. S. Alonso, M. S. Martinez, M. B. Jimenez-Diaz, S. F. Bazaga, I. Angulo-Barturen, J. N. Haselden, J. Louttit, Y. Cui, A. Sridhar, A.-M. Zeeman, C. Kocken, R. Sauerwein, K. Dechering, V. M. Avery, S. Duffy, M. Delves, R. Sinden, A. Ruecker, K. S. Wickham, R. Rochford, J. Gahagen, L. Iyer, E. Riccio, J. Mirsalis, I. Bathhurst, T. Rueckle, X. Ding, B. Campo, D. Leroy, M. J. Rogers, P. K. Rathod, J. N. Burrows, S. A. Charman, A long-duration dihydroorotate dehydrogenase inhibitor (DSM265) for prevention and treatment of malaria. *Sci. Transl. Med.* **7**, 296ra111 (2015). doi:10.1126/scitranslmed.aaa6645 Medline

17. J. B. Baell, J. W. M. Nissink, Seven Year Itch: Pan-Assay Interference Compounds (PAINS) in 2017-Utility and Limitations. *ACS Chem. Biol.* **13**, 36–44 (2018). doi:10.1021/acschembio.7b00903 Medline

18. C. K. Dong, S. Urgaonkar, J. F. Cortese, F.-J. Gamo, J. F. Garcia-Bustos, M. J. Lafuente, V. Patel, L. Ross, B. I. Coleman, E. R. Derbyshire, C. B. Clish, A. E. Serrano, M. Cromwell, R. H. Barker Jr., J. D. Dvorin, M. T. Duraisingh, D. F. Wirth, J. Clardy, R. Mazitschek, Identification and validation of tetracyclic benzothiazepines as *Plasmodium falciparum* cytochrome bc_1 inhibitors. *Chem. Biol.* **18**, 1602–1610 (2011). doi:10.1016/j.chembiol.2011.09.016 Medline

19. A. N. Cowell, E. S. Istvan, A. K. Lukens, M. G. Gomez-Lorenzo, M. Vanaerschot, T. Sakata-Kato, E. L. Flannery, P. Magistrado, E. Owen, M. Abraham, G. LaMonte, H. J. Painter, R. M. Williams, V. Franco, M. Linares, I. Arriaga, S. Bopp, V. C. Corey, N. F. Gnädig, O. Coburn-Flynn, C. Reimer, P. Gupta, J. M. Murithi, P. A. Moura, O. Fuchs, E. Sasaki, S. W. Kim, C. H. Teng, L. T. Wang, A. Akidil, S. Adjalley, P. A. Willis, D. Siegel, O. Tanaseichuk, Y. Zhong, Y. Zhou, M. Llinás, S. Ottilie, F.-J. Gamo, M. C. S. Lee, D. E. Goldberg, D. A. Fidock, D. F. Wirth, E. A. Winzeler, Mapping the malaria parasite druggable genome by using in vitro evolution and chemogenomics. *Science* **359**, 191–199 (2018). doi:10.1126/science.aan4472 Medline

20. E. L. Allman, H. J. Painter, J. Samra, M. Carrasquilla, M. Llinás, Metabolomic profiling of the malaria box reveals antimalarial target pathways. *Antimicrob. Agents Chemother.* **60**, 6635–6649 (2016). doi:10.1128/AAC.01224-16 Medline

21. H. J. Painter, J. M. Morrisey, M. W. Mather, A. B. Vaidya, Specific role of mitochondrial electron transport in blood-stage *Plasmodium falciparum*. *Nature* **446**, 88–91 (2007). doi:10.1038/nature05572 Medline

22. M. R. Luth, P. Gupta, S. Ottilie, E. A. Winzeler, Using in vitro evolution and whole genome analysis to discover next generation targets for antimalarial drug discovery. *ACS Infect. Dis.* **4**, 301–314 (2018). doi:10.1021/acsinfecdis.7b00276 Medline

23. V. Patel, M. Booker, M. Kramer, L. Ross, C. A. Celatka, L. M. Kennedy, J. D. Dvorin, M. T. Duraisingh, P. Sliz, D. F. Wirth, J. Clardy, Identification and characterization of small

molecule inhibitors of *Plasmodium falciparum* dihydroorotate dehydrogenase. *J. Biol. Chem.* **283**, 35078–35085 (2008). doi:10.1074/jbc.M804990200 Medline

24. N. A. Malmquist, R. Gujjar, P. K. Rathod, M. A. Phillips, Analysis of flavin oxidation and electron-transfer inhibition in *Plasmodium falciparum* dihydroorotate dehydrogenase. *Biochemistry* **47**, 2466–2475 (2008). doi:10.1021/bi702218c Medline

25. D. E. Hurt, J. Widom, J. Clardy, Structure of *Plasmodium falciparum* dihydroorotate dehydrogenase with a bound inhibitor. *Acta Crystallogr. D Biol. Crystallogr.* **62**, 312–323 (2006). doi:10.1107/S0907444905042642 Medline

26. X. Deng, S. Kokkonda, F. El Mazouni, J. White, J. N. Burrows, W. Kaminsky, S. A. Charman, D. Matthews, P. K. Rathod, M. A. Phillips, Fluorine modulates species selectivity in the triazolopyrimidine class of *Plasmodium falciparum* dihydroorotate dehydrogenase inhibitors. *J. Med. Chem.* **57**, 5381–5394 (2014). doi:10.1021/jm500481t Medline

27. L. S. Ross, F. J. Gamo, M. J. Lafuente-Monasterio, O. M. P. Singh, P. Rowland, R. C. Wiegand, D. F. Wirth, In vitro resistance selections for *Plasmodium falciparum* dihydroorotate dehydrogenase inhibitors give mutants with multiple point mutations in the drug-binding site and altered growth. *J. Biol. Chem.* **289**, 17980–17995 (2014). doi:10.1074/jbc.M114.558353 Medline

28. T. G. Nam, C. W. McNamara, S. Bopp, N. V. Dharia, S. Meister, G. M. C. Bonamy, D. M. Plouffe, N. Kato, S. McCormack, B. Bursulaya, H. Ke, A. B. Vaidya, P. G. Schultz, E. A. Winzeler, A chemical genomic analysis of decoquinate, a *Plasmodium falciparum* cytochrome b inhibitor. *ACS Chem. Biol.* **6**, 1214–1222 (2011). doi:10.1021/cb200105d Medline

29. V. C. Corey, A. K. Lukens, E. S. Istvan, M. C. S. Lee, V. Franco, P. Magistrado, O. Coburn-Flynn, T. Sakata-Kato, O. Fuchs, N. F. Gnädig, G. Goldgof, M. Linares, M. G. Gomez-Lorenzo, C. De Cózar, M. J. Lafuente-Monasterio, S. Prats, S. Meister, O. Tanaseichuk, M. Wree, Y. Zhou, P. A. Willis, F.-J. Gamo, D. E. Goldberg, D. A. Fidock, D. F. Wirth, E. A. Winzeler, A broad analysis of resistance development in the malaria parasite. *Nat. Commun.* **7**, 11901 (2016). doi:10.1038/ncomms11901 Medline

30. J. N. Burrows, R. H. van Huijsduijnen, J. J. Möhrle, C. Oeuvray, T. N. C. Wells, Designing the next generation of medicines for malaria control and eradication. *Malar. J.* **12**, 187 (2013). doi:10.1186/1475-2875-12-187 Medline

31. B. K. Yeung, B. Zou, M. Rottmann, S. B. Lakshminarayana, S. H. Ang, S. Y. Leong, J. Tan, J. Wong, S. Keller-Maerki, C. Fischli, A. Goh, E. K. Schmitt, P. Krastel, E. Francotte, K. Kuhen, D. Plouffe, K. Henson, T. Wagner, E. A. Winzeler, F. Petersen, R. Brun, V. Dartois, T. T. Diagana, T. H. Keller, Spirotetrahydro beta-carbolines (spiroindolones): A new class of potent and orally efficacious compounds for the treatment of malaria. *J. Med. Chem.* **53**, 5155–5164 (2010). doi:10.1021/jm100410f Medline

32. A. Ouattara, A. E. Barry, S. Dutta, E. J. Remarque, J. G. Beeson, C. V. Plowe, Designing malaria vaccines to circumvent antigen variability. *Vaccine* **33**, 7506–7512 (2015). doi:10.1016/j.vaccine.2015.09.110 Medline

33. B. Mordmüller, G. Surat, H. Lagler, S. Chakravarty, A. S. Ishizuka, A. Lalremruata, M. Gmeiner, J. J. Campo, M. Esen, A. J. Ruben, J. Held, C. L. Calle, J. B. Mengue, T. Gebru, J. Ibáñez, M. Sulyok, E. R. James, P. F. Billingsley, K. C. Natasha, A. Manoj, T. Murshedkar, A. Gunasekera, A. G. Eappen, T. Li, R. E. Stafford, M. Li, P. L. Felgner, R. A. Seder, T. L. Richie, B. K. Sim, S. L. Hoffman, P. G. Kremsner, Sterile protection against human malaria by chemoattenuated PfSPZ vaccine. *Nature* **542**, 445–449 (2017). doi:10.1038/nature21060 Medline

34. R. J. Landovitz, R. Kofron, M. McCauley, The promise and pitfalls of long-acting injectable agents for HIV prevention. *Curr. Opin. HIV AIDS* **11**, 122–128 (2016). doi:10.1097/COH.0000000000000219 Medline

35. S. Iwata, J. W. Lee, K. Okada, J. K. Lee, M. Iwata, B. Rasmussen, T. A. Link, S. Ramaswamy, B. K. Jap, Complete structure of the 11-subunit bovine mitochondrial cytochrome bc1 complex. *Science* **281**, 64–71 (1998). doi:10.1126/science.281.5373.64 Medline

36. D. Birth, W. C. Kao, C. Hunte, Structural analysis of atovaquone-inhibited cytochrome bc1 complex reveals the molecular basis of antimalarial drug action. *Nat. Commun.* **5**, 4029 (2014). doi:10.1038/ncomms5029 Medline

37. S. Yalaoui, S. Zougbédé, S. Charrin, O. Silvie, C. Arduise, K. Farhati, C. Boucheix, D. Mazier, E. Rubinstein, P. Froissard, Hepatocyte permissiveness to *Plasmodium* infection is conveyed by a short and structurally conserved region of the CD81 large extracellular domain. *PLOS Pathog.* **4**, e1000010 (2008). doi:10.1371/journal.ppat.1000010 Medline

38. A. Roth, S. P. Maher, A. J. Conway, R. Ubalee, V. Chaumeau, C. Andolina, S. A. Kaba, A. Vantaux, M. A. Bakowski, R. T. Luque, S. R. Adapa, N. Singh, S. J. Barnes, C. A. Cooper, M. Rouillier, C. W. McNamara, S. A. Mikolajczak, N. Sather, B. Witkowski, B. Campo, S. H. I. Kappe, D. E. Lanar, F. Nosten, S. Davidson, R. H. Y. Jiang, D. E. Kyle, J. H. Adams, A comprehensive model for assessment of liver stage therapies targeting *Plasmodium vivax* and *Plasmodium falciparum*. *Nat. Commun.* **9**, 1837 (2018). doi:10.1038/s41467-018-04221-9 Medline

39. C. Andolina, J. Landier, V. Carrara, C. S. Chu, J.-F. Franetich, A. Roth, L. Rénia, C. Roucher, N. J. White, G. Snounou, F. Nosten, The suitability of laboratory-bred Anopheles cracens for the production of *Plasmodium vivax* sporozoites. *Malar. J.* **14**, 312 (2015). doi:10.1186/s12936-015-0830-0 Medline

40. E. J. Lupton, A. Roth, R. Patrapuvich, S. P. Maher, N. Singh, J. Sattabongkot, J. H. Adams, Enhancing longevity of *Plasmodium vivax* and *P. falciparum* sporozoites after dissection from mosquito salivary glands. *Parasitol. Int.* **64**, 211–218 (2015). doi:10.1016/j.parint.2014.11.016 Medline

41. J. H. Zhang, T. D. Chung, K. R. Oldenburg, A simple statistical parameter for use in evaluation and validation of high throughput screening assays. *J. Biomol. Screen.* **4**, 67–73 (1999). doi:10.1177/108705719900400206 Medline

42. R. König, C. Y. Chiang, B. P. Tu, S. F. Yan, P. D. DeJesus, A. Romero, T. Bergauer, A. Orth, U. Krueger, Y. Zhou, S. K. Chanda, A probability-based approach for the analysis of large-scale RNAi screens. *Nat. Methods* **4**, 847–849 (2007). doi:10.1038/nmeth1089 Medline

43. H. Fang, J. Gough, supraHex: An R/Bioconductor package for tabular omics data analysis using a supra-hexagonal map. *Biochem. Biophys. Res. Commun.* **443**, 285–289 (2014). doi:10.1016/j.bbrc.2013.11.103 Medline

44. J. D. Johnson, R. A. Dennull, L. Gerena, M. Lopez-Sanchez, N. E. Roncal, N. C. Waters, Assessment and continued validation of the malaria SYBR green I-based fluorescence assay for use in malaria drug screening. *Antimicrob. Agents Chemother.* **51**, 1926–1933 (2007). doi:10.1128/AAC.01607-06 Medline

45. M. J. Manary, S. S. Singhakul, E. L. Flannery, S. E. R. Bopp, V. C. Corey, A. T. Bright, C. W. McNamara, J. R. Walker, E. A. Winzeler, Identification of pathogen genomic variants through an integrated pipeline. *BMC Bioinformatics* **15**, 63 (2014). doi:10.1186/1471-2105-15-63 Medline

46. A. McKenna, M. Hanna, E. Banks, A. Sivachenko, K. Cibulskis, A. Kernytsky, K. Garimella, D. Altshuler, S. Gabriel, M. Daly, M. A. DePristo, The Genome Analysis Toolkit: A MapReduce framework for analyzing next-generation DNA sequencing data. *Genome Res.* **20**, 1297–1303 (2010). doi:10.1101/gr.107524.110 Medline
